# Codonopsis radix: a review of resource utilisation, postharvest processing, quality assessment, and its polysaccharide composition

**DOI:** 10.3389/fphar.2024.1366556

**Published:** 2024-04-30

**Authors:** Wei Liang, Jiachen Sun, Gang Bai, Daiyu Qiu, Qian Li, Pengbin Dong, Yuan Chen, Fengxia Guo

**Affiliations:** ^1^ State Key Laboratory of Arid Land Crop Science, College of Agronomy, College of Life Science and Technology, Gansu Agricultural University, Lanzhou, China; ^2^ School of Biotechnology and Food Science, Tianjin University of Commerce, Tianjin, China

**Keywords:** Codonopsis radix, resource utilization, sulfur fumigation, rubbing and sweating, quality evaluation, polysaccharides

## Abstract

Codonopsis radix is the dried root of *C*. *pilosula* (Franch.) Nannf., *C. pilosula* Nannf. var. *modesta* (Nannf.) L. T. Shen, or *C. tangshen* Oliv., constitutes a botanical medicine with a profound historical lineage. It encompasses an array of bioactive constituents, including polyacetylenes, phenylpropanoids, alkaloids, triterpenoids, and polysaccharides, conferring upon it substantial medicinal and edible values. Consequently, it has garnered widespread attention from numerous scholars. In recent years, driven by advancements in modern traditional Chinese medicine, considerable strides have been taken in exploring resources utilization, traditional processing, quality evaluation and polysaccharide research of Codonopsis radix. However, there is a lack of systematic and comprehensive reporting on these research results. This paper provides a summary of recent advances in *Codonopsis* research, identifies existing issues in *Codonopsis* studies, and offers insights into future research directions. The aim is to provide insights and literature support for forthcoming investigations into *Codonopsis*.

## Graphical Abstract

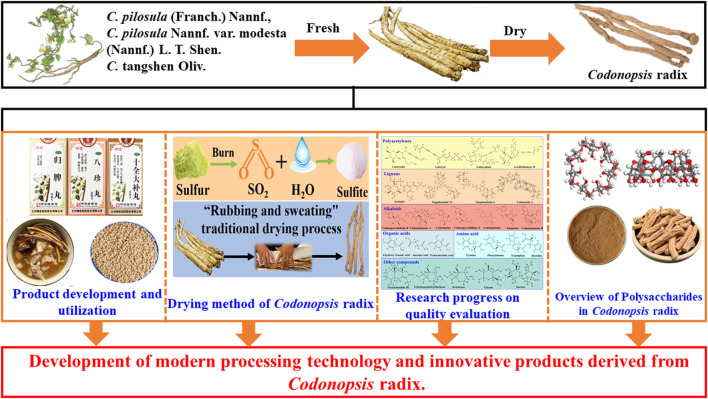

## 1 Introduction

“Dangshen,” also known as Codonopsis radix, is the common name for the dried root of *C. pilosula* (Franch.) Nannf., *C. pilosula* Nannf. var. *modesta* (Nannf.) L. T. Shen, or *C. tangshen* Oliv (Jiang et al., 2016). It has been a extensively employed traditional botanical remedy for over 2000 years in China and other Asian nations. It is associated with strengthening the immune system, improving gastrointestinal function, alleviating gastric ulcers, enhancing appetite, and reducing blood pressure (He et al., 2015). In November 2023, China’s National Health Commission and the State Administration for Market Inspection and Administration officially included Codonopsis radix in the list of substances that are both food and traditional Chinese medicine. Reportedly, the State Food and Drug Administration has approved nearly 200 health foods containing Codonopsis radix for production and sale (Gao et al., 2018a). Reportedly, the State Food and Drug Administration has approved nearly 200 health foods containing Codonopsis radix for production and sale (Gao et al., 2018b). Therefore, Codonopsis radix is a plant medicine with significant development potential and has become a focal point in modern traditional Chinese medicine research.

Codonopsis radix holds significant medicinal and nutritional value. In everyday use, it serves not only as a medicinal agent but also functions as a dietary supplement in Asian countries, including China, Japan, Korea, and Singapore. It is incorporated into the preparation of tea, alcoholic beverages, soups, and porridges (He et al., 2015; Zou et al., 2020a). The extensive applications of Codonopsis radix and the growing market demand have posed challenges for its production to meet market requirements. Moreover, given that Codonopsis radix falls under the category of rhizomatous medicinal herbs, a considerable amount of its above-ground parts is annually discarded during the harvesting process, resulting in resource wastage and environmental pollution (Yang et al., 2019b). As a result, improving the efficiency of Codonopsis radix resource utilization and undertaking research and development of associated products have emerged as central concerns in Codonopsis radix studies. Harvesting and processing procedures significantly influence the quality of Codonopsis radix. In regions where Codonopsis radix is cultivated, processing methods like sulfur fumigation and “rubbing and sweating” have been progressively established. Furthermore, some scholars have explored modern drying techniques for Codonopsis radix (Yue et al., 2023a; Yue et al., 2023b). Nonetheless, it is important to highlight that research on processing methods in Codonopsis radix-producing areas is still relatively limited and in its nascent phase. *Codonopsis* plants encompasses a diverse array of chemical components, such as sugars, flavonoids, triterpenes, steroids, alkaloids, resinous substances, and more. Despite this, there is currently an absence of a well-defined quality evaluation standard for Codonopsis radix (Gao et al., 2018b; Luan et al., 2021). Notably, the 2020 edition of the “Chinese Pharmacopoeia” mandates the evaluation of Codonopsis radix solely based on morphology, microscopy, and thin-layer chromatography. This evidently falls short of providing a comprehensive assessment of Codonopsis radix quality. *Codonopsis* plants contain a significant amount of polysaccharide components. Recent research has uncovered that *Codonopsis* polysaccharides manifest various activities, encompassing immunomodulatory effects, antioxidant and anti-aging properties, as well as anti-tumor activity (Zou et al., 2019; Wu et al., 2020a). These diverse activities imply considerable development potential, underscoring polysaccharide research as a pivotal focus in Codonopsis radix studies.

This paper offers a comprehensive and systematic review of research advancements in the utilization of *Codonopsis* resources, geographical processing, quality assessment, and other related aspects. Additionally, it provides insights into future research directions concerning Codonopsis radix. The primary objective is to provide literature support and theoretical foundations for the modernization and high-quality development of Codonopsis radix research.

## 2 Current uses of Codonopsis radix

### 2.1 Utilization as local and tradional medicine

**FIGURE 1:**
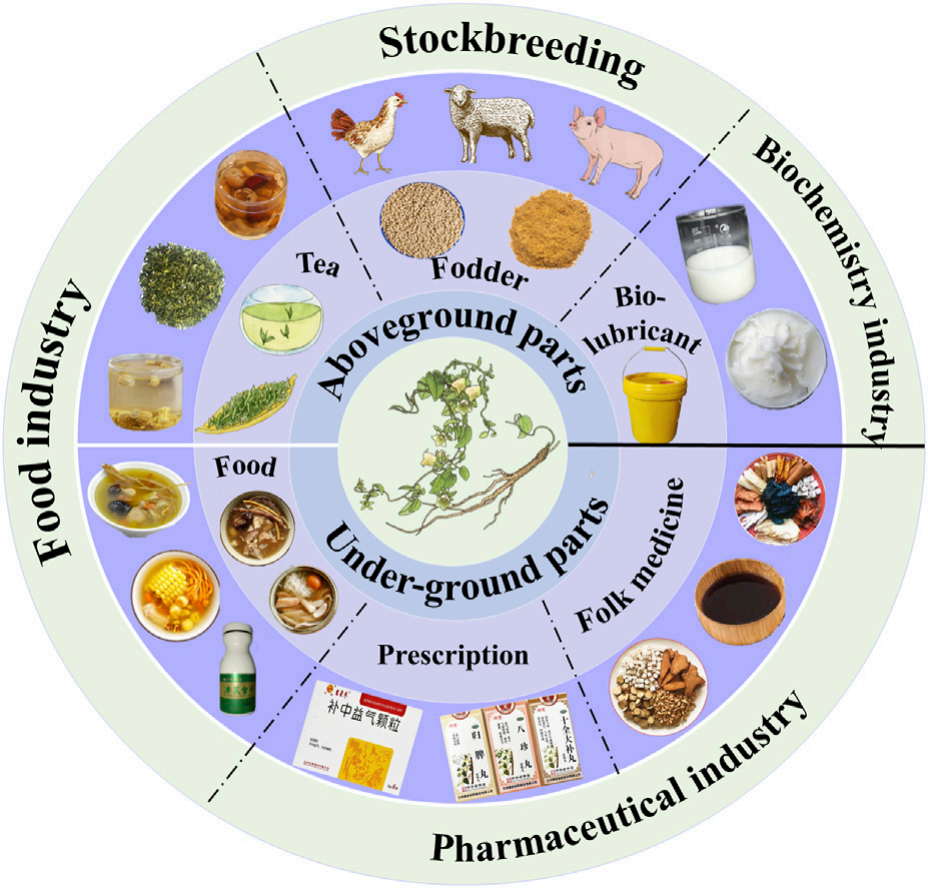
Utilization of *Codonopsis* materials.

Codonopsis radix has been widely used in traditional medicine practices worldwide for centuries. Documented as a Traditional Chinese Medicine (TCM) since the Qing Dynasty in Ben Cao Cong Xin (Gao et al., 2018b), it has been part of the Chinese Pharmacopoeia since the 1963 edition and is esteemed for its medicinal properties. Frequently, it is blended with other TCM ingredients to formulate diverse prescriptions for treating various diseases. For instance, Si-jun-zi Decoction is employed to alleviate symptoms of qi insufficiency in the middle-jiao, spleen, and stomach weakness, reduced appetite, and bowel irregularities. The Bu-zhong-yi-qi Decoction shows promise in relieving symptoms associated with spleen and qi deficiency, including chronic diarrhea and rectocele. It might also be advantageous for conditions involving stomach and uterus displacement. The Bu-fei Decoction is renowned for its capacity to strengthen the lungs and enhance qi levels, while the Sheng-mai Powder is frequently prescribed for conditions like thirst due to insufficient body fluid and qi (Gao et al., 2018b). Codonopsis radix has been employed as a traditional medicinal remedy not only within China but also across various nations for an extensive duration. In South Korea, the root of *C. pilosula* is acknowledged as the accepted species of Codonopsis radix in the Korean Herbal Pharmacopoeia and is used to treat dyspepsia, relieve fatigue, and improve the respiratory system. In the Japanese Pharmacopoeia, both the root of *C. pilosul*a and *C. pilosula* subsp. *tangshen* are recognized as species of Codonopsis radix and are widely used as traditional medicines and as raw materials in many OTC drugs. Codonopsis radix is frequently employed as a traditional botanical drug in Vietnam, exhibiting similar applications to its usage in China (Wakana et al., 2013; Gao et al., 2018b). In conclusion, Codonopsis radix shows promising medicinal potential, and conducting further comprehensive research on this plant species will significantly contribute to the advancement of novel drug development in related fields (Figure 1).

### 2.2 Utilization as food and others

The *Codonopsis* plant, known for its rich nutritional value, is widely utilized not only for medicinal purposes but also as a common food additive. However, the current development and utilization of *Codonopsis* plant resources are relatively limited, with research primarily concentrated on exploring tea, fodder, food, and chemical formulations. The specific research report is as follows (Figure 1).

1 Tea: Codonopsis radix tea is a traditional Chinese herbal tea commonly prepared with Flos Chrysanthemi, red dates, and Lycium chinensis. Tea beverages crafted from Codonopsis radix roots enjoy widespread popularity in both South Korea and China (Xiuzhi et al., 2016). Recent investigations have unveiled the presence of polyacetylenes, polyenes, flavonoids, alkaloids, steroids, terpenoids, and organic acids in the stems and leaves of Codonopsis radix, thereby indicating substantial nutritional value (Zeng et al., 2022). Consequently, certain scholars have processed the stems and leaves of Codonopsis radix to create Codonopsis leaf tea, revealing notable health-promoting effects (Yang et al., 2019c).
2 Fodder: Efforts have been made in China to explore the potential of the aboveground parts of *C. pilosula* for use as animal feed due to their nutritional value and lower cost (Yang et al., 2021). Studies have demonstrated that incorporating *C. pilosula* stems and leaves into chicken feed can significantly improve the growth performance and immunity of broilers (Ma, 2015), while adding *C. pilosula* stem and leaf to the daily feed of laying hens can effectively enhance egg quality and production rate (He et al., 1989). Similarly, the addition stems and leaves of *C. pilosula* to pig feed has been shown to markedly improve the growth performance of pigs (Dong-Zhanlian, 2014). In addition, researchers have reported that adding the extracted dregs of *C. pilosula* to the daily feed of sheep can substantially enhance the protein content of mutton (Wang, 2015). Moreover, the growth performance and resistance against foot-and-mouth disease in growing-finishing pigs have been observed to be enhanced through the addition of a combination of *A. membranaceus* and C*. pilosula* extracts referred to as HEM dietary supplementation (Cheng et al., 2020a; Cheng et al., 2020b). The vast cultivation of *C. pilosula* in China results in the harvesting of only its rhizomes for use, while its aboveground parts, such as stems and leaves, are typically discarded, causing waste and pollution. The above-ground parts of Codonopsis radix, rich in nutritional content, present substantial research potential for utilization in animal feed.
3 Food: In 2023, Codonopsis radix was approved by the National Health Commission of China and the State Administration for Market Regulation to be included in the list of plants for both medicinal and edible purposes. Owing to the presence of protein, polysaccharides, essential vitamins (such as vitamin A, B1, B2, and B3), as well as various minerals (including calcium, magnesium, iron, and zinc) in *Codonopsis* plants, they are widely employed in traditional Chinese medicinal diets (Luan et al., 2021). For instance, Codonopsis radix is a common ingredient in traditional Chinese cuisine, featuring in dishes like Codonopsis chicken soup, known for its efficacy in tonifying the middle and replenishing vital energy, and Codonopsis porridge, which aids in treating fatigue and deficiency of spleen and stomach qi (Gao et al., 2018b). Presently, the China Food and Drug Administration has sanctioned almost 200 health products containing Codonopsis radix. Some of these, including Yedao Lu Gui Jiu and Chungui Shenqiling Capsules, are believed to possess anti-fatigue properties. Additionally, in other countries such as Singapore, South Korea, and Norway, Codonopsis plants are also consumed as a functional food (Luan et al., 2021).
4 Bio-lubricant: Green synthesis and the utilization of agricultural waste for material and energy production have garnered attention due to their potential for cleaner production and sustainable development (Vazifehkhoran and Triolo, 2019). Min et al. investigated a plant wax extracted from the non-medicinal portion of *C. pilosula*, analyzing its composition, thermal stability, and lubricating properties. They proposed that this environmentally friendly biolubricant could be well-suited for applications in open systems and the food processing industry (Xie et al., 2020).

## 3 Postharvest processing

**FIGURE 2:**
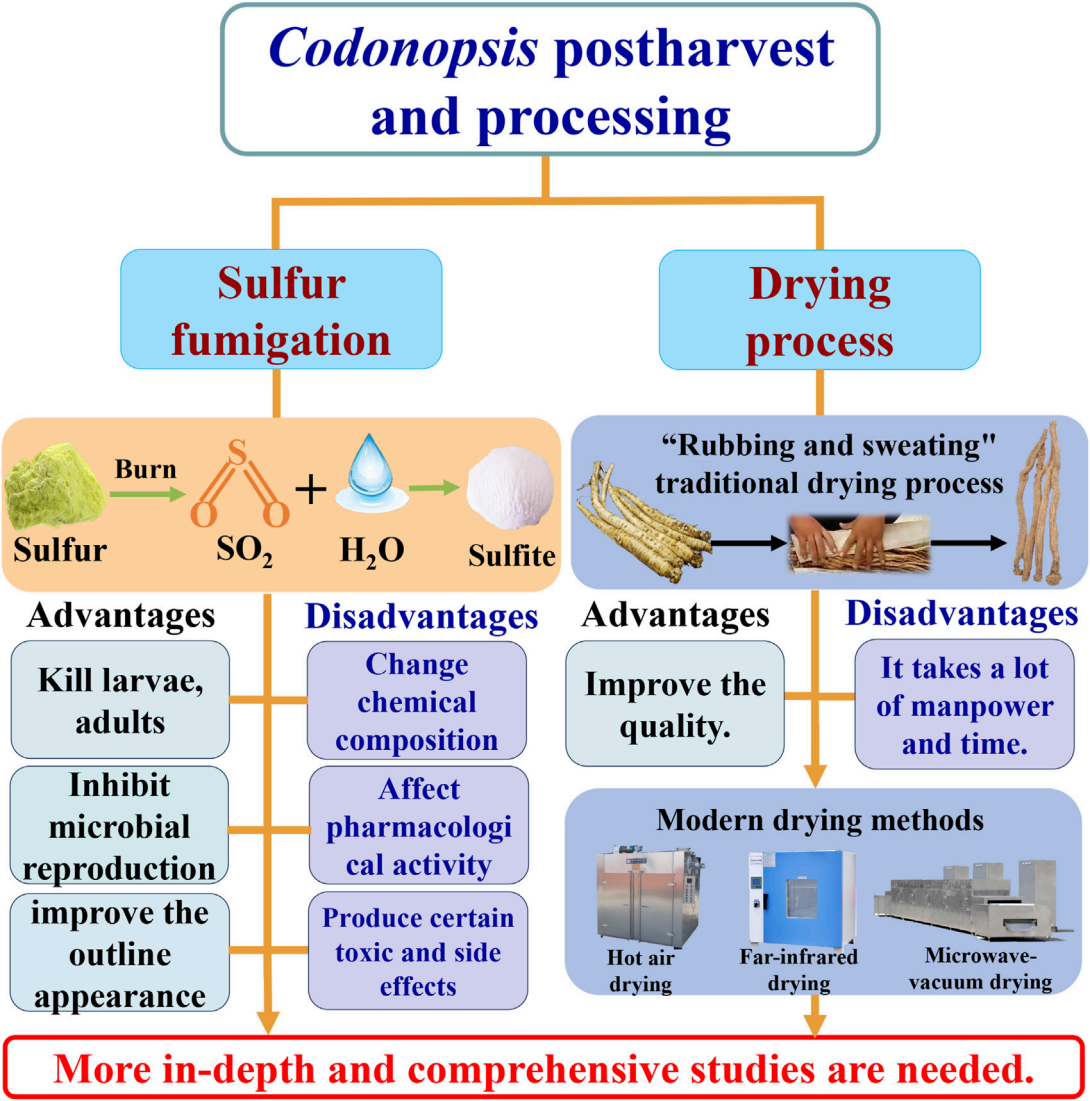
*Codonopsis* Radix postharvest processing.

Freshly harvested *C. pilosula* plants root cannot be used directly, and requires certain post-harvest treatments to facilitate preservation and improve quality. Sulfur fumigation and drying treatment are the most critical links in the harvesting and processing of Codonopsis radix (Figure 2).

### 3.1 Sulfur fumigation

The sulfur fumigation of traditional Chinese medicinal materials refers to the process in which fresh medicinal materials are placed in an enclosed space, and then sulfur is ignited within that space, facilitating thorough contact between the medicinal materials and sulfur fumes. Combustion of sulfur produces sulfur dioxide, which reacts with water molecules present in fresh botanical drugs to form sulfites. Sulfite has strong reducing power, can kill larvae, adults and eggs, and inhibit microbial reproduction; at the same time, sulfite can inhibit the activity of oxidase, slow down the browning of Chinese medicinal materials, and help improve the appearance of Chinese medicinal materials (Zhan et al., 2018). Sulfur fumigation, a long-standing traditional Chinese medicine processing technique, finds extensive application in the treatment of diverse medicinal substances due to its uncomplicated procedure and economical nature (Deng et al., 2022). There are related studies and records of sulfur fumigation in medicinal materials such as Angelicae dahuricae radix [Umbelliferae; Angelica dahurica (Fisch.ex Hoffm.)Benth.et Hook.f.] (Wang et al., 2009), Chrysanthemi flos [Compositae; *Chrysanthemum morifolium* Ramat.] (Wang et al., 2014), Dioscoreae rhizoma [Dioscoreaceae; *Dioscorea opposita* Thunb.] (Chen et al., 2017). Despite the aforementioned effects, recent research has revealed that the consumption of sulfur fumigated medicinal materials can induce adverse symptoms such as coughing and chest tightness in patients, posing a potential risk to their health and compromising the bioactivity of the metabolites, thereby diminishing the quality of these medicinal materials (Zhu et al., 2015; Jiang et al., 2020). Therefore, China, the United States and Europe have successively issued standards to limit the content of SO_2_ in TCM (Xu et al., 2020).

The Codonopsis radix possesses a rich nutrient profile and high sugar content, rendering it susceptible to insect infestation and mildew formation during storage. Consequently, sulfur fumigation is commonly employed in the production area to extend its shelf life and mitigate mildew growth. Nevertheless, to extend the storage period of Codonopsis radix and enhance its appearance, local drug farmers and dealers frequently engage in excessive sulfur fumigation. This practice alters the quality, efficacy, and safety of the medicinal material. Scholars have conducted research to investigate the impact of sulfur fumigation on Codonopsis radix. Ma et al., 2014 used ultra-high resolution quadrupole time-of-flight mass spectrometry (UHPLC UHD Q-TOF MS/MS) to compare the differences in the chemical composition of sulfur-fumigated *Codonopsis pilosula* and shade-dried *C. pilosula* samples; found that sulfur fumigation can significantly reduce atractylenolide III, codonopsine, geniposide, lobeyolin, lobeyolinin and vanillic acid content, and tryptophan sulfate, atractylenolide III sulfate, lobetylinin sulfate and other sulfated derivatives were detected at the same time, indicating that sulfur fumigation will affect the quality of *C. pilosula*. In addition, studies have found that sulfur fumigated *C. pilosula* can significantly reduce its pharmacological activities such as immune regulation and anti-fatigue (Liu et al., 2014). The above research shows that sulfur smoked Codonopsis radix will change its metabolite composition, affect its pharmacological activity, and produce certain toxic and side effects.

Due to the growing attention devoted to the issue of sulfur-fumigated medicinal materials, several researchers have undertaken investigations into the desulfurization process of Codonopsis radix subjected to sulfur fumigation Xu et al., 2019 conducted a comparative study on non-sulfur-fumigated Codonopsis radix, sulfur-fumigated Codonopsis radix and thermally desulfurized Codonopsis radix using metabolomics and glycomics, and found that the contents of Lobetyolin, baicalin, atractyenolides II and atractylenolides III in Codonopsis radix were significantly reduced after sulfur fumigation; while the content of 5-HMF was significantly increased (Xu et al., 2020). Furthermore, this study also revealed that sulfur fumigation leads to an increase in the content of Fru, Glc, Man, and GalA in Codonopsis radix, while desulfurization treatment results in a decrease in their content. Therefore, although heat desulfurization treatment may reduce the SO_2_ content in sulfur-fumigated Codonopsis radix, it alters the composition ratio of metabolites in Codonopsis radix, thereby affecting its quality (Xu et al., 2020). Zhao et al. investigated the ultrasonic desulfurization process of Codonopsis radix and identified the optimal conditions: a temperature of 50°C, an ethanol-to-Codonopsis radix ratio of 10:1, a power of 700W, and a treatment time of 50 min (Zhao and Wang, 2016). However, the mechanism underlying the impact of desulfurization treatment on the quality and pharmacological activity of Codonopsis radix remains elusive, necessitating further in-depth and comprehensive investigation.

### 3.2 Drying process

Fresh Codonopsis radix cannot be used as a medicinal material immediately after harvest; it needs to undergo a series of processes to make it suitable for use as a decoction or as raw material in Chinese medicines. In Wenxian County, Gansu Province, and Pingshun County, Shanxi Province, people have gradually developed a unique traditional processing technology of Codonopsis radix during the long-term production process. The traditional drying process of Codonopsis radix, known as “rubbing and sweating,” encompasses various steps, including drying, rubbing, and sweating. Firstly, the freshly harvested rhizomes of Codonopsis radix should be air-dried for 2–3 days until the roots attain a soft and pliable texture, enabling them to flex. Subsequently, the softened roots of Codonopsis radix should be vigorously rubbed with both hands to enhance its compactness, followed by piling and covering them with a straw mat for the sweating process. After the sweating period, which typically lasts for 2–3 days, the covering is removed and left to air dry for an additional 2–3 days. Subsequently, the dried Codonopsis radix undergoes repeated rubbing and sweating cycles until it achieves complete desiccation. Codonopsis radix which is obtained by the “rubbing and sweating” method, is highly esteemed for its exceptional texture and superior quality. Guo and Dai et al. have demonstrated that the “rubbing and sweating” drying technique effectively enhances the levels of Atractylodes III and polysaccharides in Codonopsis radix (Dai et al., 2018; Guo et al., 2018).

The selection of different drying process methods directly impacts the quality of Chinese medicinal materials. “Rubbing and sweating” as a unique production process, is applied to a variety of traditional Chinese medicine, to cause the attention of some scholars. Some scholars have found that applying the “sweating” process to Magnoliae officinalis cortex [Magnoliaceae; *Magnolia officinalis* Rehd.et Wils.] can effectively increase the content of active constituents such as magnolol and honokiol in the final medicinal product. They propose that the “sweating” process influences the composition of microbial communities, including genera such as Aspergillus, *Klebsiella*, *Enterococcus*, and *Bacillus*, in Magnoliae officinalis cortex, thereby impacting the content of metabolites (Wu et al., 2019). Additionally, some scholars conducted a metabolomics study on Salviae miltiorrhizae radix et rhizome [Lamiaceae; *Salvia miltiorrhiza* Bge] subjected to “sweating” and “non-sweating” processes. The results indicated that the “sweating” treatment significantly promoted the generation of intermediate metabolites associated with the synthesis of tanshinone and salvianolic acid in Salviae miltiorrhizae radix et rhizome. This is conducive to the accumulation of tanshinone and salvianolic acid, thereby enhancing the quality of the medicinal material (Cao et al., 2020). However, there is no report on the internal mechanism of Codonopsis radix “rubbing and sweating” to improve the quality of medicinal materials. Utilizing modern biological research methods to elucidate the internal mechanisms of the traditional processing technique involving “rubbing and sweating” for Codonopsis radix represents a crucial avenue for future research.

Due to the labor-intensive and time-consuming nature of the traditional processing method involving “rubbing and sweating” for Codonopsis radix, researchers have explored and investigated contemporary drying techniques for this botanical drug. Yue et al. conducted a comparative analysis of the drying characteristics and quality of *C. pilosula* using various methods, including hot air drying, far-infrared drying, radio frequency vacuum drying, vacuum drying, and rotary microwave vacuum drying. The research findings have demonstrated that the application of rotating microwave vacuum drying (at an ultrasonic frequency of 20 kHz, power level of 60 W, and duration of 30 min) is advantageous for preserving the content of total flavonoids, leaf casein, polysaccharides, total phenols, flavon, and syringin in Codonopsis radix (Yue et al., 2023a; Yue et al., 2023b). Zhu et al. investigated the moisture variation dynamics of Codonopsis radix under the methods of hot air drying and infrared drying. They employed the Weibull distribution function to simulate the drying process of Codonopsis radix. The outcomes revealed that the water activation energy for Codonopsis radix in hot air drying and infrared drying were 40.40 and 70.21 kJ/mol, respectively. This study furnishes data support for the establishment and optimization of the modern drying process for Codonopsis radix (Zhu et al., 2017). However, there is still a paucity of research on modern drying methods for Codonopsis radix overall, and many contemporary drying techniques remain in the experimental phase with limited application in production. The focus of future research on Codonopsis radix drying is to develop a modern drying technology that not only improves the drying efficiency but also enhances the quality of the medicinal materials.

## 4 Research for quality evaluation of Codonopsis radix

Due to non-standard market systems and market supervision and control, counterfeit and inferior Codonopsis radix products often appear. Therefore, the key to the quality control in Codonopsis radix lies in the establishment of quality analysis methods (Zhu et al., 2022). At present, the Chinese Pharmacopoeia (2020 edition) identifies Codonopsis radix from three aspects: morphology, microscopy, thin-layer chromatography, but lacks quantitative evaluation indicators. It is required that the moisture content shall not exceed 16.0%, the total ash content shall not exceed 5.0%, sulfur dioxide content shall not exceed 400 mg/kg, and the leaching content shall not be less than 55.0%. These tests are obviously not enough to evaluate the quality of Codonopsis radix accurately. This section presents a concise overview of the latest studies conducted on assessing the quality of Codonopsis radix, offering guidance for future investigations into modern analytical approaches for this botanical species.

Table 1 shows the species, chemical component, part, extraction methods and analysis technique, and other details in quantitative studies related to Codonopsis radix. With the advancement of contemporary detection and analysis technology, a multitude of techniques have been employed for the determination of Codonopsis radix’s chemical composition and assessment of its quality. These encompass high-performance liquid chromatography (HPLC) and ultra-performance liquid chromatography (UPLC) coupled with diverse detectors such as ultraviolet detector (UV), photo diode Array (PDA), diode array detector (DAD), charged aerosol detector (CAD), and evaporative light scattering detector (ELSD). DAD and PDA, have higher sensitivity and wider linear range, belong to the same type of detector, and are suitable for most metabolites with UV absorption, which have become powerful tools for qualitative and quantitative studies of Codonopsis radix and other botanical drugs. The CAD and ELSD, as complementary to the UV detector, offer advantages in sensitivity and specificity and can be employed for the detection of metabolites lacking UV chromophores (Magnusson et al., 2015). Some scholars have utilized the CAD and evaporative light scattering detector ELSD to determine the content of D-fructose, glucose, sucrose, and atractylenolide III in Codonopsis radix to assess its quality (Li et al., 2005; Xie et al., 2022). Additionally, Fourier transform infrared spectroscopy (FT-IR), ultra-performance liquid chromatography-tandem mass spectrometry (UPLC-MS/MS), and nuclear magnetic resonance (NMR) have also been utilized (Li et al., 2009; Luo et al., 2017; An et al., 2018). C_18_ column, Amide column and HP-20 column are often used for the separation and detection of metabolite in Codonopsis ginseng. However, C_18_ column is more widely used in the quality evaluation of Codonopsis radix because of its advantages of high selectivity, high sensitivity, and user-friendly operation.

**TABLE 1:** Application of modern analytical techniques in quantitative analysis of quality control of Codonopsis Radix.

No.	Species	Chemical component	Part	Extraction methods	Analysis Technique	References
1	C. *pilosula*	Lobetyolin, Syringin, Atractylenolide III	leaves, roots	1 g powder was extracted with 50/25 mL of 75%/100% methanol, ultrasonication (power: 100 W, frequency: 40 KHz) for 30/45 min (Lobetyolin/ Syringin,Atractylenolide III).	LC- MS/MS: Agilent C_18_ (2.1 mm × 100 mm, 3 μm) column at 35°C, and the flow rate was 0.3 mL/min. Mobile phase consisting of (A) 0.1% formic acid and (B) methanol. In negative ESI mode, on spray voltage, 5500 V; collision energy, 30 V; and capillary temperature, 550°C. (Lobetyolin).	Zeng et al. (2022)
					HPLC: Agela venusil ASB-C_18_ (4.6 mm × 250 mm, 5 μm) at room temperature and the flow rate was 0.8 mL/min. Mobile phase consisting of (A) 0.1% phosphoric acid solution and (B) acetonitrile. The detection wavelength was 220 nm. (Syringin, Atractylenolide III)	
2	*C. pilosula*, *C. pilosula* var. *Modesta*, *C. tangshen*	D-fructose, Glucose, Sucrose, FOS (GF2–GF6)	roots	0.1 g powder was extracted with 20 mL of 60 % methanol, ultrasonication (power: 250 W, frequency: 40 KHz) for 60 min.	HPLC-CAD: Waters XBridge™ Amide column (4.6 × 250 mm, 3.5 μm, Waters Corp.) and the flow rate was 1.0 mL/min. Mobile phase consisting of (A) acetonitrile and (B) 0.1% triethylamine aqueous solution. CAD detector: nitrogen gas pressure, 55.0 pa; data collection rate, 10 Hz; and noise filter, 5.0 s.	Xie et al. (2022)
3	*C. pilosula, C. pilosula var. Modesta, C. tangshen*	Lobetyolin, total flavonoids, pigments, total solid contents	roots	89-95% ethanol extraction	FT-NIR: Instrument resolution is specified at 4 cm^−1^. Each spectrum was acquired by averaging 64 scans across the wavenumber range of 4000–10000 cm^-1^, while background spectra were obtained against air. The samples were equilibrated to room temperature (25°C) prior to NIR spectra collecting.	Luo et al. (2017)
					HPLC: Agela venusil ASB-C18 (4.6 mm × 250 mm, 5 μm) at 30°C and the flow rate was 1.0 mL/min. Mobile phase consisting of 20% acetonitrile-water (v/v). The detection wavelength was set at 269 nm. (lobetyolin).	
					Sodium nitrite-aluminium nitrate and tartrazine colorimetric methods were used to quantify total flavonoids and pigments, respectively.	
4	*C. pilosula, C. pilosula* var. *Modesta, C. tangshen*	Lobetyolin	roots	1.0 g powder was extracted with 25 mL of 60 % methanol, ultrasonication (power: 200 W, frequency: 40 KHz) for 30 min.	HPLC: Elite C_18_ (4.6 mm × 250 mm, 5 μm) at 25°C and the flow rate was 0.8 mL/min. Mobile phase consisting of (A) 0.1% phosphoric acid solution and (B) acetonitrile. The detection wavelength was 220 nm.	Gu et al. (2016)
5	*C. pilosula, C. pilosula* var. *Modesta, C. tangshen*	Lobetyol, Lobetyolin, tangshenoside I	roots	50 mg powder was extracted with 30 mL of 70 % methanol, ultrasonication for 60 min.	HPLC-DAD: YMC-Pack Pro-C_18_-column (4.6 mm i.d. × 250 mm, 5 µm) at room temperature. and the flow rate was 1.0 mL/min. Mobile phase consisting of (A) a water-1.0 % aqueous solution of 0.1 M phosphoric acid and (B) an acetonitrile-1.0 % aqueous solution of 0.1 M phosphoric acid. The detection wavelength was 267 nm.	Kim et al. (2014)
6	*C. pilosula, C. pilosula* var. *Modesta, C. tangshen*	Codonopyrrolidium B, Codonopyrrolidium A, Tangshenoside I, Cordifolioidyne B, Lobetyolinin, Lobetyolin, Lobetyol	roots	1.0 g powder was extracted with 15 mL of methanol, ultrasonication for 20 min.	HPLC-DAD: Kromasil C_18_ column (4.6 mm × 250 mm, 5 μm) at 30 °C and the flow rate was 1.0 mL/min. Mobile phase consisting of (A) acetonitrile and (B) 0.1% phosphoric acid. The detection wavelength was 215 nm.	He et al. (2014a)
7	*C. pilosula, C. pilosula* var. *Modesta, C. tangshen*	Codonopyrrolidium B, codonopyrrolidium A, tangshenoside I, cordifolioidyne B, lobetyolinin, lobetyolin, lobetyol	roots	1.0 g powder was extracted with 15 mL of methanol, ultrasonication for 20 min.	HPLC-DAD:Kromasil C_18_ column (4.6 mm × 250 mm, 5 μm) at 30 °C and the flow rate was 1.0 mL/min. Mobile phase consisting of (A) acetonitrile and (B) 0.1% phosphoric acid. The detection wavelength was 215 nm.	He et al. (2014b)
8	*C. pilosula, C. pilosula* var. *Modesta, C. tangshen*	Codonopyrrolidum B, Histidine, 4-hydroxy benzoic acid, tangshenoside I, codonopyrrolidum A, lobetyolin, codonoside A, tetradeca-4E,8E,12E-triene-10-yne-1,6,7-triol, lobetyol	roots	sat. n-BuOH (n-BuOH pre-saturated with H_2_O)/H_2_O, collected partition layer was dried.	HPLC: Dianion HP-20 column and semi-preparative HPLC separation, at 30 °C. Mobile phase consisting of (A) acetonitrile and (B) 0.1% formic acid. The detection wavelength was 254 nm.	Lin et al. (2013)
9	*C. pilosula, C. pilosula* var. *Modesta, C. tangshen*	Lobetyolin	roots	1.0 g powder was extracted with 50 mL of methanol, ultrasonication for 60 min.	HPLC: C_18_ column (4.6 mm × 250 mm, 5 μm) at 25 °C and the flow rate was 1.0 mL/min. Mobile phase consisting of (A) acetonitrile and (B) water. The detection wavelength was 270 nm.	Qing-Wen et al. (2009)
10	*C. pilosula, C. pilosula* var. *Modesta, C. tangshen*	Codonopsine, Codonopsinine, codotubulosine A, codotubulosine B, adenosine, 5-(hydroxymethyl)furfural	roots	1.0 g powder was extracted with 10 mL of water, ultrasonication for 30 min at room temperature. (Three times)	NMR: 1H NMR spectra were recorded in methanol-d4 (99.9%) using a Varian UNITY plus 400 MHz spectrometer. For each sample, 200 scans were recorded with the following parameters: 0.187 Hz/point; spectra width, 14,400 Hz; pulse width, 4.0 μs; relaxation delay, 1 s.	Li et al. (2009)
11	*C. pilosula,*	Tangshenoside I, lobetyolin, lobetyol	roots	1.0 g powder was extracted with 25 mL of methanol, was then extracted by refluxing for 120 min.	HPLC: A Zorbax XDB RP-C_18_ column at 20 °C. Mobile phase consisting of (A) acetonitrile and (B) water. The detection wavelength was 267 and 295 nm.	Qiao et al. (2007)
12	*C. pilosula, C. pilosula* var. *Modesta, C. tangshen*	Atractylenolide III	roots	2.0 g powder was extracted with 50 mL of methanol, ultrasonication for 30 min.	HPLC: Kromasil C_18_ column (4.6 mm × 250 mm, 5 μm) and the flow rate was 0.80 mL/min. Mobile phase consisting of (A) methanol and (B) water. The detection wavelength was 220 nm.	Hao et al. (2002)
13	*C. pilosula*	Atractylenolide III	roots	5.0 g powder was extracted with 25 mL of methanol, ultrasonication for 30 min. N-Butanol extraction.	HPLC-ELSDA, HPLC-PDA: HypersilODS-2 C_18_ column (4.6 mm × 250 mm, 5 μm) and the flow rate was 1.0 mL/min. Mobile phase consisting of (A) methanol and (B) water. The detection wavelength was 220 nm.	Li et al. (2005)
14	*C. pilosula, C. pilosula* var. *Modesta, C. tangshen*	Atractylenolide III, lobetyolin	roots	0.6 g powder was extracted with 10 mL of n-hexane/ethanol, ultrasonication. The extract was evaporated to dryness and dissolved in methanol. (Atractylenolide III/ lobetyolin)	HPLC-PDA: C_18_ column (4.6 mm × 250 mm, 5 μm) at 25 °C and the flow rate was 1.0 mL/min. Mobile phase consisting of (A) acetonitrile and (B) water/0.1% acetic acid. The detection wavelength was 220/267.3 nm. (Atractylenolide III/ lobetyolin)	Pang et al. (2008)
15	*C. pilosula, C. pilosula* var. *Modesta, C. tangshen*	Lobetyolin	roots	4.0 g powder was extracted with 100 mL of methanol, soxhlet extraction for 30 min. The extract was evaporated to dryness and dissolved in methanol.	HPLC-PDA: Supelco Discovery C_18_ column (4.6 mm × 250 mm, 5 μm) at 30 °C and the flow rate was 1.0 mL/min. Mobile phase consisting of (A) acetonitrile and (B) 0.5% acetic acid. The detection wavelength was 268 nm.	Song et al. (2008)
16	*C. pilosula*	Lobetyolin	Stems, leaves, flowers	0.5 g powder was extracted with 10 mL of methanol, ultrasonication (power: 250 W, frequency: 20 KHz) for 40 min.	HPLC: Merck Purospher Star LP RP-8 endcapped (4.6 mm × 250 mm, 5 μm) at 30 °C and the flow rate was 1.0 mL/min. Mobile phase consisting of (A) acetonitrile and (B) water. The detection wavelength was 490 nm.	Cheng et al. (2020b)
17	*C. pilosula*	Lobetyolin	roots	1.0 g powder was extracted with 50 mL of methanol, ultrasonication (power: 100 W, frequency: 40 KHz) for 40 min.	HPLC-DAD: Sunfire C_18_ column (4.6 mm × 250 mm, 5 μm) at 30 °C and the flow rate was 1.0 mL/min. Mobile phase consisting of (A) methanol and (B) water. The detection wavelength was 267 nm.	Duan et al. (2012)
18	*C. pilosula, C. pilosula* var. *Modesta, C. tangshen*	Syringin, tangshenoside I, lobetyolin, atractylenolide III	roots	1.0 g powder was extracted with 25 mL of methanol, ultrasonication (power: 250 W, frequency: 59 KHz) for 30 min. The extract was evaporated to dryness and dissolved in methanol.	HPLC: InertSustainl C_18_ column (4.6 mm × 250 mm, 5 μm) at 30°C and the flow rate was 0.8 mL/min. Mobile phase consisting of (A) acetonitrile and (B) 0.2% phosphoric acid. The detection wavelength was 220 nm.	Zhang et al. (2022)
19	*C. pilosula, C. pilosula* var. *Modesta, C. tangshen*	Adenosine, Tryptophan, Syringin, tangshenoside I, Lobetyolin, Atractylenolide III	roots	2.0 g powder was extracted with 20 mL of 70% methanol, ultrasonication (power: 140 W, frequency: 42 KHz) for 30 min.	HPLC: ACE Neptune-C_18_ column (4.6 mm × 250 mm, 5 μm) at 20 °C and the flow rate was 0.8 mL/min. Mobile phase consisting of (A) acetonitrile and (B) 0.15 % formic acid. The detection wavelength was 260 nm.	Huang et al. (2022)
20	*C. pilosula, C. pilosula* var. *Modesta, C. tangshen*	Lobetyolin, syringin	roots	1.0 g powder was extracted with 50 mL of methanol, immersing 15 min and ultrasonication for 30 min. The extract was evaporated to dryness and dissolved in methanol.	HPLC: Thermo scientific C_18_ column (4.6 mm × 250 mm, 5 μm) at 25 °C and the flow rate was 1.0 mL/min. Mobile phase consisting of (A) acetonitrile and (B) water. The detection wavelength was 266 nm.	Chen et al. (2016)
21	*C. tangshen*	Lobetyolin, Atractylenolide III, Protocatechuic acid, Succinic acid, Adenosine Phenylalanine, Tyrosine, Tryptophan	roots	1.0 g powder was extracted with 25 mL of 50% methanol, ultrasonication for 60 min.	UPLC-MS: Acquity UPLC BEH-C_18_ column (2.1 mm × 100 mm, 1.7 μm) at 35 °C and the flow rate was 0.3 mL/min. Mobile phase consisting of (A) acetonitrile and (B) 0.1 % formic acid. In positive/negative ESI mode, on spray voltage, 2000 V; collision energy, 40 eV; and capillary temperature, 320°C.	An et al. (2018)
22	*C. pilosula, C. pilosula* var. *Modesta, C. tangshen*	Lobetyolin, Tangshenoside Ⅳ, Atractylenolide III.	roots	5.0 g powder was extracted with 50 mL of methanol, ultrasonication for 30 min. The extract was evaporated to dryness and dissolved in methanol. The extract was evaporated to dryness and dissolved in methanol.	HPLC: Elite SinoChrom ODS-BP C_18_ column (4.6 mm × 250 mm, 5 μm) at 25°C and the flow rate was 1.0 mL/min. Mobile phase consisting of (A) acetonitrile and (B) water. The detection wavelength was 266 nm.	Li et al. (2011)

According to the findings presented in, ultrasonic extraction emerges as the predominant technique for extracting active metabolites from Codonopsis radix owing to its inherent advantages, including superior extraction efficiency, reduced time requirements, and simplified operational procedures. Methanol aqueous solutions with varying proportions (primarily 60%–80%) serve as the principal solvent for extracting the active metabolites of Codonopsis radix. Simultaneously, most literature employs water and acetonitrile as mobile phases for quantifying Codonopsis radix, while setting the detection wavelength within the range of 220–270 nm.

**FIGURE 3:**
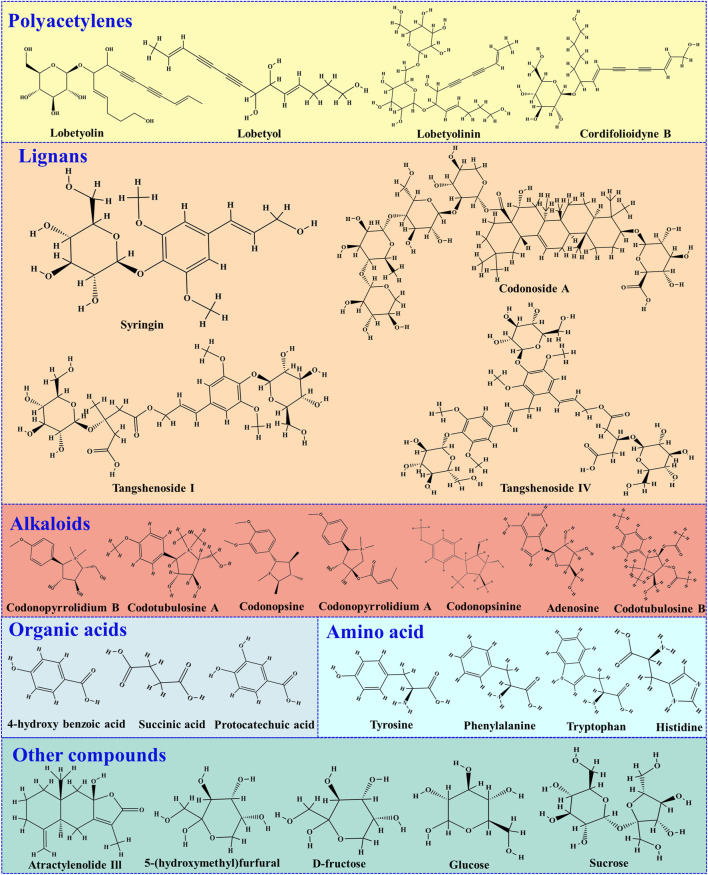
Chemical structures of some active metabolites in Codonopsis radix that can be quantitatively detected.

After a thorough review of the latest literature on quality control research of Codonopsis radix, it has been observed that despite containing hundreds of chemical substances, only around 30 have been subjected to quantitative analysis for quality control purposes. These include polyacetylenes, lignans, alkaloids, organic acids and amino acids among others (Figure 3). The bioactive metabolites Lobetyol, Lobetyolin, and Lobetyolinin are crucial polyacetylenes identified in Codonopsis radix that have demonstrated diverse pharmacological effects, encompassing antiviral, anti-inflammatory, proliferation inhibitory, and antitumor activities. (He et al., 2020; Chen et al., 2021; Xie et al., 2023). He Jing-yu et al. developed an HPLC-UV method to simultaneously determine polyacetylenes (lobetol, lobetolin, lobetollinin, cordifolioidone B), phenylpropanoid (tangshenoside I), and pyrrolidine alkaloids (codonopyrrolidins A, B) in medicinal materials of Codonopsis radix. This method was applied to differentiate various varieties of *Codonopsis*. The findings revealed that *C. Pilosula* and *C. Pilosula* var. *modesta* exhibited similar chemical compositions, while *C. tangshen* displayed significant variations in its chemical profile compared to the former two (He et al., 2014a; He et al., 2014b).

Syringin is a phenylpropanoid glycoside that exhibits a variety of pharmacological properties, including its capacity to reduce inflammation, protect against radiation damage, and inhibit the growth of cancer cells (Dong et al., 2021; Hossin et al., 2021). Atractylenolide III is a significant active metabolite found in *Codonopsis*, as well as in *Atractylodes macrocephala* Koidz*. and Chloranthus henryi* Hemsl., with a diverse range of biological activities such as anti-inflammatory, antitumor, and antiallergic responses (Coyoy-Salgado et al., 2019). Zeng et al., 2022 employed metabolomics techniques to investigate the chemical constituents of both the aerial and underground parts of *C. pilosula*, wherein syringin and Atractylolide III were identified in the stems and leaves of *C. pilosula*. The quantification of these two metabolites was accomplished using HPLC coupled with a C_18_ chromatography column at a detection wavelength of 220 nm, thereby providing robust data support for the comprehensive utilization of the aboveground portions of *C. pilosul*a.

Among the active metabolites suitable for quantitative analysis, Lobetyol, Lobetyolin, Lobetyolinin, Syringin, atractylolide III exhibit various pharmacological activities. Therefore, these metabolites have the potential to serve as markers for evaluating the quality of Codonopsis radix. Overall, the current research methods for establishing quality standards for Codonopsis radix are relatively outdated. Consequently, it is an urgent scientific imperative to elucidate the key metabolites of Codonopsis radix and develop a comprehensive determination method capable of evaluating its quality comprehensively.

## 5 Overview of polysaccharides in Codonopsis radix

*Codonopsis* polysaccharides, which are recognized as another major biologically active substance in *Codonopsis* (Zou et al., 2020a)*,* can be extracted from both the root and leaves (Li et al., 2012; Yang et al., 2019c). According to reports, the polysaccharide content in the entire *Codonopsis* plant can reach up to 6% (Luan et al., 2021). Recent studies have demonstrated that *Codonopsis* polysaccharides possess various activities such as immunomodulatory effects, antioxidant and anti-aging properties, anti-tumor activities, among others (Yang et al., 2013; Cai et al., 2014; Zou et al., 2019; Wu et al., 2020a). The significant role played by *Codonopsis* polysaccharides underscores its substantial potential for development in the utilization of *Codonopsis* resources, product development, and quality evaluation. In this section, we provide a concise overview of the current research progress on *Codonopsis* polysaccharide. The objective is to stimulate scholars’ interest in *Codonopsis* polysaccharide research and offer valuable literature data and information.

### 5.1 Extraction and purification of Codonopsis polysaccharide

Table 2 provides a compilation of polysaccharide names, sources, extracted parts, analysis techniques, extraction and purification methods, molecular weight, and other pertinent information.

**TABLE 2:** Summary of extraction and purification of polysaccharides from Codonopsis radix.

No.	Polysaccharide names	Source	Part	Analysis Technique	Extraction and Purification methods	Molecular Weight (Da)	Chemical property	Reference
1	CPPS1		root	GC, FT-IR, HPLC	WP, Ultrafiltration	—	Crude polysaccharide	Zhang et al. (2005)
2	CPPS2	*C. pilosula*	root	GC, FT-IR, HPLC	WP, Ultrafiltration	—	Crude polysaccharide	Zhang et al. (2005)
3	CPPS3		root	GC, FT-IR, HPLC	WP, Sephadex G-75 Ultrafiltration, Sephadex	1.06 × 10^6^	Acidic, purified polysaccharides	Zhang et al. (2005)
4	COP-1(1)	*C. pilosula*	root	HPLC, HPGPC	WP, Sephacryl S-200HR column and Sephadex G-25	2.10 × 10^3^	Neutral, purified polysaccharides	Liu et al. (2016)
5	COP-W1	*C tangshen*	root	FT-IR, GC–MS, NMR, HPGPC	WP, Sephacryl S-200 HR column	2.34 × 10^3^	Acidic, purified polysaccharides	Wu et al. (2020b)
6	CPP(1)	*C. pilosula*	root	GC-MS, UV, IR, NMR	WP, DEAE-Sepharose CL-6B, Sephadex G-75 column	1.10 × 10^4^	Neutral, purified polysaccharides	Sun and Liu (2008)
7	CPP (2)	*C. pilosula*	root	Capillary Electrophoretic Method	WP,	No	Crude polysaccharide	Yang et al. (2008)
8	CPP (3)	*C. pilosula*	root	GC	WP,	No	Crude polysaccharide	Zhang et al. (2012)
9	CPP (4)	*C. pilosula*	root	HPLC, HPGPC	WP, DEAE Cellulose-52 column	2.15 × 10^4^	Crude polysaccharide	Li et al. (2016)
10	CPP(5)	*C. pilosula*	root	HPSEC-RID, FT-IR NMR, HPLC	WP,	4.63 × 10^7^ 5.69 × 10^4^	Crude polysaccharide	Feng and Zhang (2020)
11	CPP1	*C. pilosula*	root	HPSEC-RID, FT-IR NMR, HPLC	DEAE Cellulose-52 column	5.692 × 10^4^	Neutral, purified polysaccharides	Feng and Zhang (2020)
12	CP1-2-1	*C tangshen*	root	HPGPC, TLC	WP, DEAE Sepharose Fast Flow column, Sephadex G-100 (separation and purification)	25.80 × 10^4^	Acidic, purified polysaccharides	Meng et al. (2020)
13	CP3-1-1	*C tangshen*	root	HPGPC, TLC	WP, DEAE Sepharose Fast Flow column, Sephadex G-100(separation and purification)	49.50 × 10^4^	Acidic, purified polysaccharides	Meng et al. (2020)
14	DSPS	*C. pilosula* (*Exopolysaccharides of endophyte*)	root	GC-MS; HPLC	WP, GC-MS	1.68 × 10^4^	Neutral, purified polysaccharides	Chen et al. (2018)
15	CPSP-1	*C. pilosula*	stem	GC-MS, NMR	WP, DEAE cellulose column	1.31 × 10^4^	Acidic, purified polysaccharides	Zou et al. (2020b)
16	CTSP-1	*C. tangshen*	stem	GC-MS, NMR	WP, ultrafiltration, DEAE cellulose column	2.30 × 10^4^	Acidic, purified polysaccharides	Zou et al. (2020b)
17	CPPS	*C. pilosula*	root	SEM, FT-IR, HPGPC, HPLC, NMR	WP, DEAE-52 and Sephadex G-200 columns	5.30 × 10^3^–2.31 × 10^5^	Neutral, purified polysaccharides	Gao et al. (2020)
18	CPS	*C. pilosula*	root	HPLC, TLC, UV	WP, DEAE cellulose column	—	Crude polysaccharide	Li (2007)
19	CPS-1	*C. pilosula*	root	HPLC, TLC, UV	WP, DEAE cellulose column	2.50 × 10^3^	Neutral, purified polysaccharides	Li (2007)
20	CPS-2	*C. pilosula*	root	HPLC, TLC, UV	WP, DEAE cellulose column	—	Acidic, purified polysaccharides	Li (2007)
21	CPS-3	*C. pilosula*	root	HPLC, TLC, UV	WP, DEAE cellulose column	1.15 × 10^5^	Acidic, purified polysaccharides	Li (2007)
22	CPS-4	*C. pilosula*	root	HPLC, TLC, UV	WP, DEAE cellulose column	>2.00 × 10^6^	Acidic, purified polysaccharides	Li (2007)
23	CPS-5	*C. pilosula*	root	HPLC, TLC, UV	WP, DEAE cellulose column	—	Acidic, purified polysaccharides	Li (2007)
24	CPP-1	*C.pilosula*	root	FT-IR, GC-MS, NMR	WP, DEAE cellulose column	7.50 × 10^4^	Neutral, purified polysaccharides	Ye et al. (2005)
25	COP-1(2)	*C. tangshen*	root	HPGPC, HPLC, FT-IR, NMR	WP, DEAE cellulose column	6.31 × 10^3^	Neutral, purified polysaccharides	Han et al. (2005)
26	COP-2(1)	*C. tangshen*	root	HPGPC, HPLC, FT-IR, NMR	WP, DEAE cellulose column	7.09 × 10^3^	Acidic, purified polysaccharides	Han et al. (2005)
27	AP1	*C.pilosula*	root	UV, AFM, GC, HPLC, FT-IR	WP, CTAB extraction method	9.47 × 10^3^	Acidic, purified polysaccharides	Ren (2008)
28	PP2	*C.pilosula*	root	UV, AFM, GC, HPLC, FT-IR	WP, CTAB extraction method	9.72 × 10^3^	Purified polysaccharides	Ren (2008)
29	CERP1	*C.pilosula*	root residue	HPAEC, HPLC, HPSEC, FT-IR, NMR, TEM	WP, DEAE-32 cellulose column	4.840 × 10^3^	Neutral, purified polysaccharides	Liu et al. (2018)
30	COP-1	*C.pilosula*	Root	HPGPC, HPLC, GC-MS	WP, DEAE-52 cellulose column	1.12 × 10^5^	Purified polysaccharides	Wang (2013)
31	COP-2	*C.pilosula*	root	HPGPC, HPLC, GC-MS	WP, DEAE-52 cellulose column	2.89 × 10^3^	Purified polysaccharides	Wang (2013)
32	COP-3	*C.pilosula*	root	HPGPC, HPLC, GC-MS	WP, DEAE-52 cellulose column	1.86 × 10^6^	Purified polysaccharides	Wang (2013)
33	COP-4	*C.pilosula*	root	HPGPC, HPLC, GC-MS	WP, DEAE-52 cellulose column	1.31 × 10^6^	Acidic, purified polysaccharides	Wang (2013)
34	RCNP	*C. pilosula*	root	HPGPC, HPLC, GC-MS, NMR	WP, DEAE-650M, Superdex G-200	1.14 × 10^4^	Neutral, purified polysaccharides	Sun et al. (2019)
35	RCAP-1	*C. pilosula*	root	HPGPC, HPLC, GC-MS, NMR	Toyopearl DEAE 650 M column	5.09 × 10^4^	Acidic, purified polysaccharides	Sun et al. (2019)
36	RCAP-2	*C. pilosula*	root	HPGPC, HPLC, GC-MS, NMR	Superdex G-200	2.58 × 10^5^	Acidic, purified polysaccharides	Sun et al. (2019)
37	RCP	*C. pilosula*	root	HPGPC,	WP, HPGPC	1.71 × 10^4^	Crude polysaccharide	Deng et al. (2019)
38	CPPF	*C. pilosula* var. *modesta,*	root	GC, HMDS, TMS,HPLC, NMR	WP, DEAE-Sepharose	2.81 × 10^3^	Neutral, purified polysaccharides	Fu et al. (2018)
39	Fructan 1, 2, 3	*C. pilosula*	root	NMR, MALDI-TOF MS, HPGPC	WP, ultrasonic extraction	2400; 2700; 3500	Acidic, purified polysaccharides	Li et al. (2018)
40	CP-A	*C. pilosula*	root	HPGPC, NMR, TOF-MS	WP, Ultrafiltration	3.60 × 10^3^	Neutral, purified polysaccharides	Li et al. (2017)
41	CP-B	*C. pilosula*	root	HPGPC, HPLC, ESI-MS, NMR	WP, ultrafiltration membrane	1.70 × 10^3^	Purified polysaccharides	Li et al. (2020)
42	CPPA	*C. pilosula*	root	GC, HPGPC	WP, DEAE	4.20 × 10^3^	Acidic, purified polysaccharides	Xin et al. (2012)
43	CPPN	*C. pilosula*	root	GC-MS, NMR, SEC-MALLS	WP, DEAE-Sepharose	2.29 × 10^3^	Neutral, purified polysaccharides	Zou et al. (2021)
44	CTPN	*C. tangshen*	root	GC-MS, NMR, SEC-MALLS	WP, DEAE-Sepharose	3.95 × 10^3^	Neutral, purified polysaccharides	Zou et al. (2021)
45	RCPO	*C. pilosula*	root	—	WP, Crude polysaccharide	—	Neutral, purified polysaccharides	Bai et al. (2020)
46	CPO	*C. pilosula*	root	HPGPC, GC–MS, FT-IR, ESI-MS, NMR	WP, DEAE-Sepharose,	3.18 × 10^2^	Crude polysaccharide	Bai et al. (2020)
47	CERP	*C. pilosula*	root	HPGPC, HPLC, RID	WP, DEAE-Sepharose	2.02 ×10^6^ 7.30 ×10^3^	Crude polysaccharide	Tang et al. (2021)
48	S-CPPA1	*C. pilosula*	stem	HPGPC, HPLC, RID, GC-MS,	DEAE cellulose and Sepharose CL-6B	1.33 × 10^5^	Acidic, purified polysaccharides	Li et al. (2012)
49	S-CPPB	*C. pilosula*	Stem	HPGPC, HPLC, RID, GC-MS,	DEAE cellulose	—	Acidic, purified polysaccharides	Li et al. (2012)
50	S-CPPC	*C. pilosula*	Stem	HPGPC, HPLC, RID, GC-MS,	DEAE cellulose	—	Acidic, purified polysaccharides	Li et al. (2012)
51	CPP-2-1	*C. pilosula*	root	HPGPC, UV, HPLC,	DEAE-cellulose and Sepharose CL-6B	1.70 × 10^5^	Acidic, purified polysaccharides	Zhu-Rui (2013)
52	CPP-2-2	*C. pilosula*	root	HPGPC, UV, HPLC,	DEAE-cellulose and Sepharose CL-6B	6.00 × 10^5^	Acidic, purified polysaccharides	Zhu-Rui (2013)
53	CPP-3-1	*C. pilosula*	root	HPGPC, UV, HPLC,	DEAE-cellulose and Sepharose CL-6B	—	Acidic, purified polysaccharides	Zhu-Rui (2013)
54	CPP-3-2	*C. pilosula*	root	HPGPC, UV, HPLC,	DEAE-cellulose and Sepharose CL-6B	14.00 × 10^5^	Acidic, purified polysaccharides	Zhu-Rui (2013)
55	CPs	*C. pilosula* var. *modesta,*	root	HPLC, UV	WP,	—	Acidic, purified polysaccharides	Chen (2007)
56	50WCP-II-I	*C. pilosula* var. *modesta,*	root	GC-MS, NMR	50 °C extracts, ion exchange chromatography (ANX Sepharose 4 Fast Flow) and gel filtration (Superdex 200 prep grade column), ultrafiltration	7.16 × 10^4^	Acidic, purified polysaccharides	Zou et al. (2014)
57	50WCP-II-Ia	*C. pilosula* var. *modesta,*	root	GC-MS, NMR	Same as above.	1.73 × 10^4^	Acidic, purified polysaccharides	Zou et al. (2014)
58	100WCP-II-I	*C. pilosula* var. *modesta,*	root	GC-MS, NMR	50 °C Residue, 100 °C extracts. ion exchange chromatography and gel filtration	5.32 × 10^4^	Acidic, purified polysaccharides	Zou et al. (2014)
59	100WCP-II-Ia	*C. pilosula* var. *modesta,*	root	GC-MS, NMR	Same as above.	1.73 × 10^4^	Acidic, purified polysaccharides	Zou et al. (2014)
60	100WCP-II-Ib	*C. pilosula* var. *modesta,*	root	GC-MS, NMR	Same as above.	1.30 × 10^3^	Acidic, purified polysaccharides	Zou et al. (2014)
61	CPP1a	*C. pilosula* var. *modesta,*	root	HPLC, HPGPC, GC–MS, IR	anion exchange chromatography (DEAE-cellulose 52)	1.01 × 10^5^	Acidic, purified polysaccharides	Bai et al. (2018)
62	CPP1b	*C. pilosula*	root	HPGPC, HPLC, GC, UV, NMR, TEM	anion exchange chromatography (DEAE-cellulose)	1.45 × 10^5^	Acidic, purified polysaccharides	Yang et al. (2013)
63	CPP1c	*C. pilosula*	root	HPGPC, GC-MS, IR, NMR, SEM	anion exchange chromatography (DEAE-cellulose 52)	1.26 × 10^5^	Acidic, purified polysaccharides	Zhang et al. (2017)
64	WCP-I	*C. pilosula* var. *modesta,*	root	GC	anion exchange chromatography (DEAE-cellulose 52)	1.67 × 10^4^	Acidic, purified polysaccharides	Zou et al. (2019)

No: WP: Water-extraction and alcohol-precipitation method.

The method of hot water extraction has been extensively employed for obtaining crude polysaccharides from Codonopsis radix. In recent years, this approach has successfully isolated a variety of polysaccharides with physiological activities from Codonopsis radix (Wu et al., 2020b; Gao et al., 2020; Meng et al., 2020). However, the hot water extraction method exhibits several limitations, including suboptimal extraction efficiency, prolonged extraction duration, challenging control over extraction conditions, and potential structural damage to polysaccharides. To overcome these constraints in the extraction of *Codonopsis* polysaccharides, various alternative methods have been employed: ultrasonic-assisted extraction (Li et al., 2018), enzyme-assisted extraction (Yuan et al., 2020), microwave-assisted extraction (Yu et al., 2011), accelerated solvent extraction (Zou et al., 2014), and subcritical water extraction (Zhang et al., 2013). Ji Hai-Yu et al. conducted a study to examine how the yield of *Codonopsis* polysaccharides is affected by varying levels of ultrasonic power during the process of ultrasonic-assisted extraction. Their findings demonstrated that an optimal range of ultrasonic power between 200 and 360 W resulted in increased polysaccharide yield, while exceeding 360 W significantly decreased the yield of *Codonopsis* polysaccharides (Ji et al., 2022). This suggests that higher ultrasonic power could damage the polysaccharide structure and promote degradation (Yang et al., 2019a).

Enzyme-facilitated extraction has attracted considerable interest owing to its mild extraction conditions, brief duration, minimal energy usage, and cost-efficiency. Gao et al., 2018a conducted a comprehensive investigation into the extraction parameters for obtaining polysaccharides from *C. pilosula* through the utilization of enzyme-assisted extraction techniques. The researchers determined that an enzyme dosage of 0.2%, a pH value of 4.2, an enzymatic hydrolysis temperature of 50°C, and a hydrolysis time of 1.5 h resulted in an extraction efficiency of 25.23% for *Codonopsis* polysaccharides.

The microwave exhibits remarkable penetrating power, exceptional selectivity, and high heating efficiency. Yu et al., 2011 have reported that the utilization of microwaves in the extraction process significantly reduces extraction time and enhances the efficiency of polysaccharide extraction from *C. pilosula*. Optimal conditions for this procedure include a microwave extraction temperature of 70°C, a duration of 25 min, a radiation power of 600 W, and a solid-liquid ratio of 1:40 (g/mL).

Accelerated solvent extraction is a high-pressure, high-temperature extraction method that utilizes an organic solvent (Giergielewicz-Mozajska et al., 2001). Zou et al., 2014 utilized this method to extract polysaccharides from *C. pilosula* Nannf. var. modesta L.T.Shen and obtained several purified polysaccharides, namely 50WCP-II-I, 50WCP-II-Ia, 100WCP-II-I, 100WCP-II-Ia, and 100WCP-II-Ib.

The subcritical water extraction method is a technique that involves heating water under high pressure to a critical state of 100°C–374°C to facilitate extraction. Zhang et al., 2012 optimized the subcritical water extraction process of *C. pilosula* and found that a solid-to-liquid ratio of 12 mL/g, extraction temperature of 150°C, and extraction time of 45 min significantly improved the extraction rate of *C. pilosula* and *C. tangshen* polysaccharides (Zhang et al., 2013). Nonetheless, there is a scarcity of studies focusing on modern technologies for extracting *Codonopsis* polysaccharides. Exploring an economical and efficient extraction method represents a crucial direction for future research in this domain.

The crude polysaccharide of Codonopsis radix is usually separated and purified by anion exchange chromatography (DEAE-Cellulose, DEAE-Cellulose 52, DEAE-Sepharose CL-6B and DEAE-Sepharose FF, etc.) and gel permeation chromatography (Sephadex G, Sephacryl S and Sepharose CL, etc.). During the separation and purification process of crude polysaccharides from Codonopsis radix, varying concentrations of eluates were employed to separate polysaccharides with distinct properties. Neutral polysaccharides are obtained by eluting with distilled water, while acidic polysaccharides are obtained by eluting with various concentrations of salt solutions. Ultimately, various gradient eluates were collected, subjected to dialysis, concentrated, and lyophilized, resulting in the isolation of distinct types of *Codonopsis* polysaccharides.

### 5.2 The physicochemical properties and biological activities of *Codonopsis* polysaccharides

The physicochemical characterization, and bioactivity evaluation of *Codonopsis* polysaccharides have been a prominent research focus in recent years. We summarize some studies on the biological activities and structural characteristics of *Codonopsis* polysaccharides in Supplementary Table S1. To date, a total of 64 polysaccharides have been successfully isolated and identified from various parts of *C. pilosula*, *C. pilosula* var. *modesta*, and *C. tangshen*, comprising 12 crude, 15 neutral, and 34 acidic polysaccharides (The acid-base properties of the remaining three polysaccharides remain undetermined.).

Currently, 34 acidic polysaccharides have been isolated from Codonopsis radix. Among these, 4 are derived from the aerial parts of *C*. *pilosula*, 17 from the roots of *C. pilosula*, and 5 from the roots of *C. tangshen*. Additionally, 8 kinds have been extracted from the roots of *C. pilosula* var. *modesta*. The majority of acid polysaccharides are isolated and purified from the roots of *Codonopsis*. The first purified pectic polysaccharide, CPP1b, has a backbone consisting of (1 → 4)-linked *α*-D-GalpA and (1 → 4)-linked *α*-D-GalA*p*6Me. It also contains rare interspersed (1 → 2)-linked *β*-L-Rh*ap*, (1, 2, 6)-linked *α*-D-Galp, and terminal α-L-Arap (Yang et al., 2013). The MTT assay was employed to assess the inhibitory rate of CPP1b on the growth of human lung adenocarcinoma A549 cells at concentrations of 0, 50, 100, 200, and 400 μg/mL. The results revealed significant cytotoxicity of CPP1b on human lung adenocarcinoma A549 cells, with an inhibition rate ranging from 46.8% to 65.4% at 48 h. These findings suggest the potential of CPP1b as a promising antitumor agent or adjuvant drug (Yang et al., 2013). Sun et al., 2019 isolated and purified two acidic polysaccharides, namely RCAP-1 and RCAP-2, from the roots of *C. pilosula*, and characterized their structural features and activities. The findings revealed that RCAP-1 and RCAP-2 were pectic polysaccharides with molecular weights of 5.09 × 10^4^ and 2.58 × 10^5^ Da, respectively. RCAP-1 and RCAP-2 were pectin-type polysaccharides with high methyl-esterification, featuring long homogalacturonan regions interspersed with a short rhamnogalacturonan I (RG-I) region. The RG-I region contained side chains comprising (1→2)-linked Rha residues attached to the O-4 position of rhamnose. The MTS assay was employed to assess the immunomodulatory activity of RCAP-1 and RCAP-2 on macrophages. The results demonstrated that the incubation of RAW264.7 macrophages with RCAP-1 and RCAP-2 (40 μg/mL) for 24 h significantly promoted the production of NO (Sun et al., 2019).

Wu et al. isolated an acidic polysaccharide, COP-W1, with a molecular weight of 2.34 × 10^4^ Da from *Codonopsis tangshen* Oliv using a hot water extraction method. Structural analysis revealed that the backbone of COP-W1 is comprised of (1→3)-linked-galactose, (1→3, 6)-linked-mannose, (1→4)-linked-mannose, and (1→6)-linked-mannose residues with branching occurring at O-3. The branches contain (1→2, 6)-linked-galactose, (1→6)-linked-galactose, and (1→4)-linked-galactose residues, terminating with α-galactose (Wu et al., 2020b). The chemical DPPH scavenging ability of COP-W1 was evaluated at concentrations of 0, 0.15, 0.3, 0.6, 1.2, 2.4, and 4.8 mg/mL. The results revealed a significant increase in the chemical DPPH scavenging ability of COP-W1 with the rise in concentration, reaching 82.9% at 2.4 mg/mL, and an IC_50_ of 0.610 mg/mL (Wu et al., 2020b).

Bai et al. isolated a water-soluble acidic polysaccharide, CPP1a, from *C. pilosula* Nannf var. *modesta* (Nannf.) L.T. Shen, with a molecular weight of 1.01 × 10^5^ Da. The results of physical and chemical property measurements indicate that CPP1a was consisted of linkages→1) -L-Rha-(4→,→1)-Ara-(5→,→1)-d-Gal-(4→,→1)-d-Gal-(6→, terminal-d-Glc at the molar ratio of 1:12:1:10:3 (Bai et al., 2018). Following a 48-h incubation of HepG2 cells with 50, 200, and 400 μg/mL CPP1a, CPP1a was observed to alter the morphology of HepG2 cells, impede cell migration, and induce cell cycle arrest in the G2/M phase. Concurrently, HepG2 cells exhibited anticancer effects by upregulating Bcl-2 and Bax, adjusting the Bax/Bcl-2 ratio, and activating caspase-3 to promote apoptosis (Bai et al., 2018).

Only 4 acidic polysaccharides have currently been isolated and purified from the stems of *C. pilosula*. Li et al., 2012 extracted crude polysaccharides from the stems of *C. pilosula* using a water extraction and alcohol precipitation method. The extracted polysaccharides were then separated into S-CPPA, S-CPPB, and S-CPPC fractions using a DEAE cellulose column. Subsequently, S-CPPA1, a purified polysaccharide, was obtained from S-CPPA and its physical and chemical properties, as well as biological activities, were evaluated. The findings revealed that S-CPPA1 is a branched polysaccharide consisting of five glucose linkage forms, namely (1→4)-linked Glcp (residue A), (1→6)-linked Galp (residue B), (1→2,6)-linked Glcp (residue C), (1→5)-linked Arc (residue D), and non-reducing terminal (1→)-linked Glcp (residue E). Furthermore, the protective impact of S-CPPA1 against renal ischemia/reperfusion (I/R) injury was assessed through the oral administration of 10 mg/kg S-CPPA1 for ten consecutive days in adult male Wistar rats, followed by renal I/R injury. The findings indicated a significant reduction in BUN, Cr, TNF-α levels, and the activities of LDH and AST in the serum of Wistar rats following oral administration of S-CPPA1. This suggests that S-CPPA1 exhibits a distinct renal protective effect (Li et al., 2012). Zou et al. employed water extraction, alcohol precipitation, and ultrafiltration methods to extract pectin-type polysaccharides from *C. pilosula* and *C. tangshen*. They successfully purified CPSP-1 and CTSP-1 with molecular weights of 1.23 and 0.1 kDa, respectively, from the stems of these plants. The structure elucidation results revealed that CPSP-1 and CTSP-1 are pectic polysaccharides consisting of long homogalacturonan regions (HG) with methyl-esterified galacturonic acid units and rhamnogalacturonan I (RG-I) regions. In addition, CTSP-1 possesses both arabinogalactan type I (AG-I) and type II (AG-II) side chains, whereas CPSP-1 only contains AG-II side chains (Zou et al., 2020b). In the context of H_2_O_2_-stimulated oxidative stress in porcine intestinal epithelial cells, incubation with stems of CPSP-1 and CTSP-1 at concentrations of 5, 10, and 20 μg/mL for 24 h resulted in a significant reduction in cellular levels of MDA, ROS, and LDH. Conversely, the enzyme activities and mRNA expressions of GSH-Px, SOD1, CAT, and T-AOC, as well as the gene expression of ZO-1, exhibited a marked increase in a dose-dependent manner (Tang et al., 2021).

Currently, 15 neutral polysaccharides have been isolated from *C. pilosula*, including 12 from its roots, 2 from *C. tangshen* roots, and 1 from the roots of *C. pilosula* var. *modesta*. RCNP is a neutral polysaccharide isolated from the roots of *C. pilosula*, with a molecular weight of 1.14 × 10^4^ Da (Sun et al., 2019). It mainly consists of a significant amount of Arabinose (Ara) and a smaller amount of Galactose (Gal). The molar ratio of these two residues is approximately 3:1. Structural analysis revealed that RCNP is a neutral polysaccharide consisting of arabinan and arabinogalactan (AG) regions. The arabinan region is composed of a main chain consisting of (1 → 5)-linked Araf residues, with single Araf residues branching at the O-3 position. The AG region is composed of (1 → 4)-, (1 → 6)-, or (1 → 3)-linked Galp residues, with branches primarily at the O-3 position of the (1 → 6)-linked Galp or O-6 position of the (1 → 3)-linked Galp residues, terminated by Araf residues (Sun et al., 2019). Liu et al., 2018 isolated a neutral polysaccharide named CERP1 from *C. pilosula* waste, with a molecular weight of 4.840 × 10^3^ Da. The structural determination results revealed that CERP1 consisted of arabinose, glucose, and galactose. The main linkage patterns identified were 1-linked β-D-glucose, 1,3-linked β-D-glucose, 1,6-linked β-D-glucose, 1,3,6-linked β-D-galactose, and 1,3,5-linked α-L-arabinose, which were the predominant linkages in CERP1. Additionally, the hypoglycemic effects of CERP1 were assessed in both *in vitro* and *in vivo* settings using insulin-treated INS-1 cells and T2DM mouse models. Treating INS-1 cells with CERP1 at concentrations of 0.2, 0.4, 0.6, and 0.8 mg/mL for 48 h effectively alleviated streptozotocin (3 mmol/L)-induced cell damage. It resulted in reduced MDA content, increased SOD activity, and improved insulin secretion. In T2DM mice, the administration of doses of 150, 300, and 600 mg/kg for 28 days led to significant decreases in fasting blood glucose, QUICKI index, TG, TC, LDL/HDL, and MDA levels. Concurrently, the activities of SOD, T-AOC, CAT, GSH-Px, and the homeostasis model assessment of insulin resistance (HOMA-IR) index were significantly increased. These findings suggest that CERP1 demonstrates robust anti-hyperglycemic activity both *in vitro* and *in vivo*, and holds the potential for development as a hypoglycemic drug or functional food (Liu et al., 2018).

Fu et al., 2018 optimized the extraction process of polysaccharides from *C. pilosula* var. *modesta* and successfully separated and purified an inulin-type fructan named CPPF from the crude polysaccharides. The structural analysis revealed that CPPF has a repeating unit composed of α-d-Glcp-(1→2)-[β-d-Fru f-(2→1)-β-d-Fru f]n-(2→1)-β-d-Fru f. CPPF, along with two commercially available prebiotics (P95s and OraftiHP), was added to the culture medium at a concentration of 1 g/L as carbon sources. This mixture was then incubated with six *Lactobacillus* species (BSS1, BS15, BS10, BSGP201683, LGG, and Hjg8) for 24 h. Following incubation, CPPF exhibited varying degrees of growth promotion among the six *Lactobacillus* species and decreased the pH of the medium, suggesting its potential prebiotic activity (Fu et al., 2018).

Zou et al., 2021 extracted a neutral polysaccharide called CTPN from *C. tangshen.* Structural identification revealed that CTPN is an inulin-type fructan linked by β-(2,1). Incubating porcine jejunal epithelial cells (IPEC-J2) with CTPN at concentrations of 5, 10, and 20 μg/mL for 24 h revealed a significant increase in the activities of T-AOC, GSH-Px, SOD, and CAT. Concurrently, it led to a decrease in the levels of MDA and LDH, ultimately enhancing the antioxidant capacity of IPEC-J2 cells.

In summary, polysaccharides constitute important active metabolic products in Codonopsis radix. However, current research on *Codonopsis* polysaccharides faces the following shortcomings: 1) The predominant method for extracting *Codonopsis* polysaccharides relies on hot water extraction, which exhibits low extraction efficiency, thereby constraining the industrial application of *Codonopsis* polysaccharides. Therefore, the application of modern extraction methods to enhance the extraction efficiency of *Codonopsis* polysaccharides holds significant importance. 2) Present research on *Codonopsis* polysaccharides is largely confined to the experimental stage, with limited studies focusing on their practical applications. Future research should emphasize the development of relevant products. 3) Investigations into *Codonopsis* polysaccharides predominantly focus on the roots, with limited exploration of polysaccharides in other *Codonopsis* plant parts. Therefore, non-medicinal parts of the *Codonopsis* plant show significant potential for polysaccharide development.

## 6 Conclusion and prospects

*C. pilosula* (Franch.) Nannf., *C. pilosula* Nannf. var. *modesta* (Nannf.) Shen, and *C*. *tangshen* Oliv. are recorded in the Chinese Pharmacopoeia and serve as the primary source plants for Codonopsis radix in traditional Chinese medicine. While these three varieties are distributed in Asia, North America, Europe, and other regions, China stands out as the main production area and the principal distributing country for Codonopsis radix. In recent years, driven by an increase in living standards and growing health consciousness, the market demand for Codonopsis radix has been steadily rising. Despite numerous scholars conducting research on *Codonopsis* resource utilization, origin processing, and quality evaluation, there are still some shortcomings.

Firstly, *Codonopsis* possesses abundant nutritional substances, not only holding medicinal value but also being developed for applications in tea, food, and fodder. However, the current research on the medicinal and edible value of Codonopsis radix is not sufficiently profound, and the development of related products falls short of meeting the demands of the modern traditional Chinese medicine market. In future research, it is imperative to increase investment in the research and development of *Codonopsis*-related products, enhance the added value of *Codonopsis* products, and create greater economic benefits.

Secondly, the “rubbing and sweating” method is a unique processing technique developed by the *Codonopsis* producing regions over a long-term production process. *Codonopsis* processed through “rubbing and sweating” has gained widespread recognition in the market due to its superior quality. However, there is no reported research on the intrinsic mechanism by which the “rubbing and sweating” method enhances the quality of medicinal materials. Therefore, elucidating the mechanism by which “rubbing and sweating” improves the quality of *Codonopsis* medicinal materials and developing a modern drying technology that can enhance both drying efficiency and medicinal material quality becomes a key focus in the processing of *Codonopsis* in its producing regions.

Thirdly, in recent years, with the advancement of detection technologies, various techniques such as HPLC, NMR, UPLC-MS/MS, FT-IR have been employed for chemical analysis in *Codonopsis*. However, the current research methods for *Codonopsis* quality standards are relatively outdated, and the Chinese Pharmacopoeia still lacks quantitative evaluation indicators for *Codonopsis*. Therefore, elucidating the key metabolites of *Codonopsis*, establishing a comprehensive determination method capable of assessing its quality comprehensively, becomes a pivotal direction for future research in *Codonopsis* quality evaluation.

Fourthly, *Codonopsis* polysaccharides exhibit various activities such as immunomodulating, anti-tumor, antioxidant, and prebiotic activities, making them significant metabolites of *Codonopsis*. However, the current extraction of *Codonopsis* polysaccharides is primarily based on hot water extraction, which has low efficiency. Consequently, improving the extraction efficiency of *Codonopsis* polysaccharides through modern extraction methods is a pressing issue in the field. Furthermore, the existing research on *Codonopsis* polysaccharides predominantly focuses on polysaccharides extracted from the roots, with limited exploration on the extraction and purification of polysaccharides from the aboveground parts. Therefore, future research should emphasize the development of polysaccharides extracted from the aboveground parts of *Codonopsis.*

This paper provides a comprehensive review of the research progress in the utilization of *Codonopsis* resources, traditional processing methods, quality evaluation, and polysaccharide studies. The aim is to offer literature support for future endeavors in the utilization of *Codonopsis* resources, development of processing techniques, and the exploration of related derivative products.
