# Supplementary material for: Codonopsis radix: a review of resource utilisation, postharvest processing, quality assessment, and its polysaccharide composition
Source: Front Pharmacol. 2024 Apr 30;15:1366556. doi: 10.3389/fphar.2024.1366556 (PMC11091420; doi:10.3389/fphar.2024.1366556)
Supplement: Supplementary file 1 [file Table1.docx]

**Table S1.** Summary of biological activities and structural characteristics of polysaccharides from Codonopsis radix.

| No. | Polysaccharide  names | Source | Biological  activities | In vitro/In vivo | Testing Subjects | Doses/Duration | Effects/Mechanisms | Structural characteristics (for instance, backbone and monosaccharide  composition) | Reference |
| --- | --- | --- | --- | --- | --- | --- | --- | --- | --- |
| 1 | CPP(1) | *C. pilosula* | Immunomodulating activity | In vitro | Mouse splenocytes induced by ConA- or LPS | 0 (control) ,50, 100, 200 μg/mL for 72 h. | stimulate lymphocyte proliferation. | D-Gal: D-Rha: D-Ara=1.12:1.00:1.12  →3)-β-D-Galp-(1→3)-α-D-Rhap-(1→ | (Sun and Liu, 2008) |
| 2 | CPP (3) | *C. pilosula* | Immunomodulating activity | In vitro | Mouse spleen B lymphocytes; mouse macrophages | 50, 100, 200, 400 μg/mL for 3days. | Enhance lymphocyte proliferation and macrophage phagocytic ability. | Glc:Gal:Xyl:Rha:Ara:GalA=102.7:7.9:2.2:1.8:1.1:1.0 | (Zhang et al., 2012) |
| 3 | RCNP | *C. pilosula* | Immunomodulating activity | In vitro | Mouse macrophage RAW264.7 cells, | 0, 1.6, 8, 40, 200, 1000 μg/mL for 24h. | No obvious cytotoxicity; | Ara: Gal=75.2:24.8(Area %)  Ara residues mainly exist as (1 → 5)-linked arabinofuranosyl (Araf) residues; the presence of galactan composed of (1 → 4)-, (1 → 6)-, and (1 → 3, 6)-linked Galp. | (Sun et al., 2019) |
| 4 | RCAP-1 | *C. pilosula* | Immunomodulating activity | In vitro | Mouse macrophage RAW264.7 cells, | 0, 1.6, 8, 40, 200, 1000 μg/mL for 24h. | No obvious cytotoxicity; enhanced the NO release. | Rha: Ara:GalA=5.7:3.5:90.8 (Area %)  GalA residues are the main glycosyl residues, GalAp mainly exist as (1 → 4)-linked GalAp. | (Sun et al., 2019) |
| 5 | RCAP-2 | *C. pilosula* | Immunomodulating activity | In vitro | Mouse macrophage RAW264.7 cells | 0, 1.6, 8, 40, 200, 1000 μg/mL for 24h. | No obvious cytotoxicity; enhanced the NO release. | Rha: Ara: GalA=3.3:3.0:93.7(Area %)  RCAP-2 were similar with those of RCAP-1. However, RCAP-2 has a high molecular weight and long α-(1 → 4)-linked GalAp backbone chain. | (Sun et al., 2019) |
| 6 | RCP | *C. pilosula* | Immunomodulating activity | In vivo | BALB/c mice; Peripheral blood lymphocytes of mice | In vivo: 100, 500 mg / kg for 15 days. | Beneficial for maintaining CD4^+^ /CD8^+^, Th1/Th2 and Treg/Th17 cell balance. | Total sugar 95.41%; Uronic acid 7.13. | (Deng et al., 2019) |
| 7 | CPO | *C. pilosula* | Immunomodulating activity | In vitro | RAW264.7 cells | 0, 0.25, 0.50, 0.75, 1.0 mg/mL for 24 h. | Improve the proliferation and phagocytic rate of RAW264.7 cells, and promote TNF- α、 IL-6 and NO production; promote phosphorylation of p38, ERK1/2, and JNK. | glucose: fructose=1.21:1  →1-*α*-D-Glcp, →2)-*β*-D-Fruf-1(→and *β*-D-Fruf- (2→. | (Bai et al., 2020) |
| 8 | 50WCP-II-Ia | *C. pilosula* var. *Modesta,* | Immunomodulating activity | In vitro | Complement fixation assay | - | complement fixation activity; ICH_50_ was 6.5 μg/mL. | Ara: Rha:Fuc:Xyl:Man:Gal:Clc: ClcA: GalA=6.9:2.9:1.7:1.2:5.7:21.8:6.3:3.0:50.6 | (Zou et al., 2014) |
| 9 | 100WCP-II-Ia | *C. pilosula* var. *Modesta,* | Immunomodulating activity | In vitro | Complement fixation assay | - | complement fixation activity; ICH_50_ was 12.2 μg/mL. | Ara: Rha:Fuc:Xyl:Man:Gal:Clc: ClcA: GalA=8.0:3.5:1.6:2.1:6.6:20.2:6.0:2.8:49.2 | (Zou et al., 2014) |
| 10 | CPP1c | *C. pilosula* | Immunomodulating activity | In vitro /In vivo | mouse splenocytes; SAMP8 mice | In vitro: 0,50, 100, 200 μg/mL for 48h;  In vivo: 200mg/kg for 14 days. | In vitro: induced proliferation of spleen cells and stimulated the secretion of IL-2, TNF-α, and IFN-γ; In vivo: positive effect on CD4^+^ /CD8^+^ T cells, CD4^+^ /CD152^+^, promote the expression of CD28, PI3K, and p38MAPK proteins. | Rha:Ara:Gal:GalA =2.99:1.15:1.94:33.29  →1)-α-l-Rhap-(2,4→, →1)-α-L-Araf-(5→, →1)-α-D-Galp-(6→ and →1)-α-D-GalpA-(4→ | (Zhang et al., 2017) |
| 11 | WCP-I | *C. pilosula* var. *Modesta,* | Immunomodulating activity | In vivo | C3H/HeJ mice | 100 mg/kg for 10 days. | Beneficial for spleen index, stimulating secretion of IL-6, TGF-β, TNF-α, and promoting sIgA production. | Ara:Rha:Man:Gal:Glc:GalA=5.5:6.4:0.7:17.6:0.2:69.6  HG backbone with RG-I region, AG-I and AG-II side-chains | (Zou et al., 2019) |
| 12 | DSPS | *C. pilosula（Exopolysaccharides of endophyte）* | Anti-tumor activity | In vitro | RAW264.7 cells, MDA-MB-231 cells | 0, 0.2, 1.0, 5.0 mg/mL for 24 or 48h. | Enhanced the TNF-αand NO release (5.0 mg/mL); promoted RAW264.7 cell migration (1.0 and 5 mg/mL); induce cell death (5 mg/mL); affects spindle orientation and positioning in cancer cells (5 mg/mL). | galactose and glucose, rhamnose, fucose, arabinose, mannose. | (Chen et al., 2018) |
| 13 | CPS | *C. pilosula* | Anti-tumor activities | In vivo | Kunming mice | 0.5 g/mL polysaccharide solution by gavage (three times a day) for 7d | Prolong the survival days of mice with ascites tumor cells. | — | (Li, 2007) |
| 14 | CPPA | *C. pilosula* | Anti-tumor activity | In vitro | The human ovarian cancer cell line HO-8910 | 25, 50, 100, and 200 μg /mL for 48 h. | Inhibited HO-8190 cell proliferation, cell migration and CD 44 protein expression. | Rha, Ara, Gala,Man, and Galacturonic acid= 1.34:3.49:1.07:0.45:1.98 | (Xin et al., 2012) |
| 15 | CPP-2-1 | *C. pilosula* | Anti-tumor activity | In vitro | HCT116 cells | 0.5, 2.5, 5 mg/mL for 72h. | Inhibiting cancer cell proliferation. | Rha:GalA:Ara:Gal=5.00:53.50:22.80:17.80 | (Zhu-Rui, 2013) |
| 16 | CPP-2-2 | *C. pilosula* | Anti-tumor activity | In vitro | HCT116 cells | 0.5, 2.5, 5 mg/mL for 72h. | Inhibiting cancer cell proliferation. | Rha:GalA:Ara:Gal=2.70:81.30:7.00:6.10 | (Zhu-Rui, 2013) |
| 17 | CPP-3-1 | *C. pilosula* | Anti-tumor activity | In vitro | HCT116 cells | 0.5, 2.5, 5 mg/mL for 72h. | Inhibiting cancer cell proliferation. | Rha:GalA:Ara:Gal=7.50:25.30:36.00:31.20 | (Zhu-Rui, 2013) |
| 18 | CPP-3-2 | *C. pilosula* | Anti-tumor activity | In vitro | HCT116 cells | 0.5, 2.5, 5 mg/mL for 72h. | Inhibiting cancer cell proliferation. | Rha:GalA:Ara:Gal=9.90:54.40:21.70:14.00 | (Zhu-Rui, 2013) |
| 19 | CPP1a | *C. pilosula* var. *Modesta,* | Anti-tumor activity | In vitro | Human hepatoma HepG2 cells | 50, 100, 200, 400 μg/mL for 48h. | Promoting HepG2 cell apoptosis and aspase-3 protein expressions. | →1)-β‑L‑Rhap‑(4→,→1)-β‑Arap‑(5→,→1)-β‑D‑GalpA‑(4→,→1)-β‑D‑Galp‑(6→,terminal‑β‑D‑Glcp | (Bai et al., 2018) |
| 20 | CPP1b | *C. pilosula* | Anti-tumor activity | In vitro | Human lung adenocarcinoma A549 cells | 0,50, 100, 200, 400 μg/mL for 48h. | Reduce cell proliferation rate. | Rha: Ara:Gal:GalA)=0.25:0.12:0.13:2.51  1,4-D-α-D-Gal*p*A;1,4-D α-D-Gal*p*A6Me; 1,2-D-β-L-Rha*p*, 1,2,6-α-D-Gal*;* α-L-Ara*p*. | (Yang et al., 2013) |
| 21 | COP-W1 | *C tangshen* | Antioxidant activities | In vitro | Chemical DPPH scavenging ability | 0, 0.15,0.3, 0.6, 1.2, 2.4, and 4.8 mg/mL. | Exhibited the strong DPPH radical scavenging activity the IC_50_ of 0.610 mg/mL. | (1→6)-linked-Man residues, (1, 3→6)-linked-Man residues, (1→4)-linked-Man residues, and (1→3)-linked-Gal residues. The branch chain included (1, 2→6)-linked-Gal residues, and (1→6)-linked-Gal residues, (1→4)-linked-Gal residues; the terminal residue was α-Gal.  Man: Rha:Glc:Gal=20.32:1.00:1.27:36.13 | (Wu et al., 2020) |
| 22 | CPP(5) | *C. pilosula* | Antioxidant and anti-tumor activities | In vitro | ABTS, DPPH and Metal ion; A2780 and Skov3 cells | ABTS and Metal ion: 1, 2, 4, 8 10 mg/mL;  A2780 and Skov3 cells: 6.25, 12.5, 25, 50, 100 μg/mL for 12h. | Enhance ABST and metal ion clearance rate, inhibit A2780 cell proliferation, and induce Skov3 cell apoptosis | — | (Feng and Zhang, 2020) |
| 23 | CPSP-1 | *C. pilosula* | Antioxidant activities | In vitro | IPEC-J2 cells | 20, 10 and 5 μg/ mL for 24h. | No cytotoxicity (20 μg/mL); increase the cell viability and cellular T-AOC; promote the clearance of cellular ROS; Enhancing cellular antioxidant enzyme activity (GSH-Px, SOD and CAT). | Ara:Rha:Gal:GalA =8.9:9.3:11.0:70.1 | (Zou et al., 2020) |
| 24 | CTSP-1 | *C. tangshen* | Antioxidant activities | In vitro | IPEC-J2 cells | 20, 10 and 5 μg/ mL for 24h. | No cytotoxicity (20 μg/mL); increase the cell viability and cellular T-AOC, promote the clearance of cellular ROS; Enhancing cellular antioxidant enzyme activity (GSH-Px, SOD and CAT). | Ara:Rha:Gal:GalA:Glc:GlcA=8.2:11.2:18.9:61.3 | (Zou et al., 2020) |
| 25 | CPPN | *C. pilosula* | Antioxidant activities and prebiotic activities | In vitro | IPEC-J2 cells; Lactobacillus bacterial strains | 5, 10, 20 μg/mL  for 24 h. | Enhanced cell antioxidant enzyme (GSH-Px, SOD, CAT) activity and reduced MDA and LDH content; the promotion of beneficial bacteria growth. | Inulin-type fructan structure, above all,  CPPN were composed of mainly terminal-Fruf, terminal-Glcp, and 2,1-linked Fruf, different molar ratios of 1.4:6.0: 92.7.  →α-D-Glcp-(1→2)→[*β*-D-Fruf-(2→1)*-β*-D-Fruf]_n1_→(2→1)*-β*-D-Fruf | (Zou et al., 2021) |
| 26 | CTPN | *C. tangshen* | Antioxidant activities and prebiotic activities | In vitro | IPEC-J2 cells; Lactobacillus bacterial strains | 5, 10, 20 μg/mL  for 24 h. | Enhanced cell antioxidant enzyme (GSH-Px, SOD, CAT) activity and reduced MDA and LDH content; the promotion of beneficial bacteria growth. | Inulin-type fructan structure, above all  CTPN were composed of mainly terminal-Fruf, terminal-Glcp, and 2,1-linked Fruf, different molar ratios of 2.5: 4.7: 92.8.  →α-D-Glcp-(1→2)→[*β*-D-Fruf-(2→1)*-β*-D-Fruf]_n2_→(2→1)*-β*-D-Fruf | (Zou et al., 2021) |
| 27 | CP1-2-1 | *C tangshen* | Anti-inflammatory activity | In vitro | Raw 264.7 cells | 0.01, 0.1, 1.0, 10, 100 μg/mL for 48h. | Inhibiting the formation and release of IL-6, TNF-α inflammatory factors | fructose | (Meng et al., 2020) |
| 28 | CP3-1-1 | *C tangshen* | Anti-inflammatory activity | In vitro | Raw 264.7 cells | 0.01, 0.1, 1.0, 10, 100 μg/mL for 48h. | Inhibiting the formation and release of IL-6, TNF-α inflammatory factors | Arabinose, rhamnose, galactose and galacturonic acid | (Meng et al., 2020) |
| 29 | CERP | *C. pilosula* | Anti-inflammatory activity | In vivo | DSS-induced colitis mice | 600 mg/kg for 7 days. | Positive effect on DAI score, histological changes; SOD, IL-10, and AhR; negative effect on MDA, IL-1 β, TNF- α, IL-6 and IL-22. | CERP was composed of mannose, rhamnose, galacturonic acid, glucose, galactose, and arabinose at the molar ratio of 1.00:3.26:27.87:10.87:7.70:9.94. | (Tang et al., 2021) |
| 30 | CP-A | *C. pilosula* | Anti-gastric ulcer activity | In vivo | Ethanol-Induced Acute Gastric Ulcer Rats | 12.5, 25, and 50 mg/kg for 7days. | Reduce gastric mucosal ulcer index and MDA, NO content; Improve the activity of MPO, SOD, and GSH-Px. | Inulin-type fructan structure, above all | (Li et al., 2017) |
| 31 | CPPF | *C. pilosula* var. *Modesta,* | Prebiotic activity | In vitro | Lactobacillus bacterial strains | - | The promotion of beneficial bacteria growth and the reduction of the culture medium's pH value demonstrate potential probiotic activity. | α-D-Glc*p*-(1 → 2) -[β-D-Fru*f*-(2 → 1)-β-D-Fru*f*]_n_-(2 → 1)-β-d-Fru*f* | (Fu et al., 2018) |
| 32 | Fructan 1, 2, 3 | *C. pilosula* | Prebiotic activity | In vitro | Bifidobacterium longum | 2.0 mg/mL for 12, 36, 60 h. | Probiotic activity is demonstrated by stimulating the growth of Bifidobacterium longum. | α-D-Glc*p*-(1 → 2) -[β-D-Fru*f*-(2 → 1)-β-D-Fru*f*]_n_-(2 → 1)-β-d-Fru*f*, (n = 15, 21, 30, respectively) | (Li et al., 2018) |
| 33 | S-CPPA1 | *C. pilosula* | Renoprotective activity | In  vivo | Kidney I/R injury of rats | 10 mg/kg for 10 days. | Reduced urea nitrogen, creatinine, and TNF-α levels; lowered LDH and AST activity; and exerted a positive impact on organizational change. | Glc: Gal: Ara = 10.5:3.4:1.7;  (1→4)-linked Glcp, (1→6)-linked Galp, (1→2,6)-linked Glcp, (1→5)-linked Araf, and non-reducing terminal (1→)-linked Glcp. | (Li et al., 2012) |
| 34 | CERP1 | *C.pilosula* | Hypoglycemic activity | In vitro/In vivo | INS-1 cells subjected to STZ-induced damage; HFD/STZ induced T2DM mice | In vitro :200, 400, 600, 800 μg/mLfor 48 h;  In vivo: 150, 300, and 600  mg/kg for 28 day | In vitro: the cell viability and insulin secretion ability were enhanced;In vivo: HOMA-IR index increases; levels of TG, TC, LDL/HDL decrease; Increased activity of T-AOC, SOD, CAT, and GSH Px. | Arabinose, Glucose, Galactose=1.00:19.83:6.94 | (Liu et al., 2018) |

# **References:**

Bai, R.B., Li, W.Y., Li, Y.D., Ma, M., Wang, Y.P., Zhang, J., et al. (2018). Cytotoxicity of two water-soluble polysaccharides from Codonopsis pilosula Nannf. var. modesta (Nannf.) LTShen against human hepatocellular carcinoma HepG2 cells and its mechanism. *International Journal of Biological Macromolecules* 120**,** 1544-1550. doi: 10.1016/j.ijbiomac.2018.09.123.

Bai, R.B., Zhang, Y.J., Jia, X.S., Fan, J.M., Hou, X.H., Wang, Y.P., et al. (2020). Isolation, characterization and immunomodulatory activity of oligosaccharides from Codonopsis pilosula. *JOURNAL OF FUNCTIONAL FOODS* 72. doi: 10.1016/j.jff.2020.104070.

Chen, M., Li, Y.Y., Liu, Z., Qu, Y.J., Zhang, H.J., Li, D.W., et al. (2018). Exopolysaccharides from a Codonopsis pilosula endophyte activate macrophages and inhibit cancer cell proliferation and migration. *Thoracic Cancer* 9(5)**,** 630-639. doi: 10.1111/1759-7714.12630.

Deng, X.L., Fu, Y.J., Luo, S., Luo, X., Wang, Q., Hu, M.H., et al. (2019). Polysaccharide from Radix Codonopsis has beneficial effects on the maintenance of T-cell balance in mice. *Biomedicine & Pharmacotherapy* 112. doi: 10.1016/j.biopha.2019.108682.

Feng, G., and Zhang, X.F. (2020). Production of a codonopsis polysaccharide iron complex and evaluation of its properties. *INTERNATIONAL JOURNAL OF BIOLOGICAL MACROMOLECULES* 162**,** 1227-1240. doi: 10.1016/j.ijbiomac.2020.06.210.

Fu, Y.P., Li, L.X., Zhang, B.Z., Paulsen, B.S., Yin, Z.Q., Huang, C., et al. (2018). Characterization and prebiotic activity in vitro of inulin-type fructan from Codonopsis pilosula roots. *Carbohydrate Polymers* 193**,** 212-220. doi: 10.1016/j.carbpol.2018.03.065.

Li, J.K., Wang, T., Zhu, Z.C., Yang, F.R., Cao, L.Y., and Gao, J.P. (2017). Structure Features and Anti-Gastric Ulcer Effects of Inulin-Type Fructan CP-A from the Roots of Codonopsis pilosula (Franch.) Nannf. *Molecules* 22(12). doi: 10.3390/molecules22122258.

Li, J.K., Zhang, X., Cao, L.Y., Ji, J.J., and Gao, J.P. (2018). Three Inulin-Type Fructans from Codonopsis pilosula (Franch.) Nannf. Roots and Their Prebiotic Activity on Bifidobacterium longum. *MOLECULES* 23(12). doi: 10.3390/molecules23123123.

Li, R.-y. (2007). *Studies on Extraction Technology of Radix Codonopsis Water-soluble Polysaccharides.* 硕士, Shanxi Medical University.

Li, Z.T., Zhu, L.B., Zhang, H., Yang, J., Zhao, J., Du, D.W., et al. (2012). Protective effect of a polysaccharide from stem of Codonopsis pilosula against renal ischemia/reperfusion injury in rats. *Carbohydrate Polymers* 90(4)**,** 1739-1743. doi: 10.1016/j.carbpol.2012.07.062.

Liu, W., Lv, X., Huang, W.H., Yao, W.B., and Gao, X.D. (2018). Characterization and hypoglycemic effect of a neutral polysaccharide extracted from the residue of Codonopsis Pilosula. *CARBOHYDRATE POLYMERS* 197**,** 215-226. doi: 10.1016/j.carbpol.2018.05.067.

Meng, Y., Xu, Y.-j., Zhang, B.-h., Chang, C., Zheng, G.-h., and Wu, Y. (2020). Study on the Anti-inflammatory Activity and Mechanism of Different Components from Codonopsis Radix Polysaccharides. *China Pharmacy* 31(11)**,** 1348-1352. doi: 10.19540/j.cnki.cjcmm.20200204.402.

Sun, Q.L., Li, Y.X., Cui, Y.S., Jiang, S.L., Dong, C.X., and Du, J. (2019). Structural characterization of three polysaccharides from the roots of Codonopsis pilosula and their immunomodulatory effects on RAW264.7 macrophages. *International Journal of Biological Macromolecules* 130**,** 556-563. doi: 10.1016/j.ijbiomac.2019.02.165.

Sun, Y.X., and Liu, J.C. (2008). Structural characterization of a water-soluble polysaccharide from the roots of Codonopsis pilosula and its immunity activity. *International Journal of Biological Macromolecules* 43(3)**,** 279-282. doi: 10.1016/j.ijbiomac.2008.06.009.

Tang, S., Liu, W., Zhao, Q.Q., Li, K.D., Zhu, J.Y., Yao, W.B., et al. (2021). Combination of polysaccharides from Astragalus membranaceus and Codonopsis pilosula ameliorated mice colitis and underlying mechanisms. *Journal of Ethnopharmacology* 264. doi: 10.1016/j.jep.2020.113280.

Wu, Q.N., Luo, M., Yao, X.D., and Yu, L. (2020). Purification, structural characterization, and antioxidant activity of the COP-W1 polysaccharide from Codonopsis tangshen Oliv. *Carbohydrate Polymers* 236. doi: 10.1016/j.carbpol.2020.116020.

Xin, T., Zhang, F.B., Jiang, Q.Y., Chen, C.H., Huang, D.Y., Li, Y.J., et al. (2012). The inhibitory effect of a polysaccharide from Codonopsis pilosula on tumor growth and metastasis in vitro. *International Journal of Biological Macromolecules* 51(5)**,** 788-793. doi: 10.1016/j.ijbiomac.2012.07.019.

Yang, C.X., Gou, Y.Q., Chen, J.Y., An, J., Chen, W.X., and Hu, F.D. (2013). Structural characterization and antitumor activity of a pectic polysaccharide from Codonopsis pilosula. *CARBOHYDRATE POLYMERS* 98(1)**,** 886-895. doi: 10.1016/j.carbpol.2013.06.079.

Zhang, P., Hu, L.H., Bai, R.B., Zheng, X.P., Ma, Y.L., Gao, X., et al. (2017). Structural characterization of a pectic polysaccharide from Codonopsis pilosula and its immunomodulatory activities in vivo and in vitro. *International Journal of Biological Macromolecules* 104**,** 1359-1369. doi: 10.1016/j.ijbiomac.2017.06.023.

Zhang, Y.-j., Liang, Z.-y., and Zhang, L.-x. (2012). Study on composition and immunological activities of crudepolysaccharide isolated from the Codonopsis pilosula. *Journal of Northwest A & F University(Natural Science Edition)* 40(07)**,** 199-202+208. doi: 10.13207/j.cnki.jnwafu.2012.07.014.

Zhu-Rui (2013). *Analyses and Anti-tumor Activitives of the Polysaccharides from Codonopsis pillosula.* 硕士, Northeast Normal University.

Zou, Y.F., Chen, X.F., Malterud, K.E., Rise, F., Barsett, H., Inngjerdingen, K.T., et al. (2014). Structural features and complement fixing activity of polysaccharides from Codonopsis pilosula Nannf. var. modesta L.T.Shen roots. *Carbohydrate Polymers* 113**,** 420-429. doi: 10.1016/j.carbpol.2014.07.036.

Zou, Y.F., Zhang, Y.Y., Fu, Y.P., Inngjerdingen, K.T., Paulsen, B.S., Feng, B., et al. (2019). A Polysaccharide Isolated from Codonopsis pilosula with Immunomodulation Effects Both In Vitro and In Vivo. *Molecules* 24(20). doi: 10.3390/molecules24203632.

Zou, Y.F., Zhang, Y.Y., Paulsen, B.S., Rise, F., Chen, Z.L., Jia, R.Y., et al. (2020). Structural features of pectic polysaccharides from stems of two species of Radix Codonopsis and their antioxidant activities. *International Journal of Biological Macromolecules* 159**,** 704-713. doi: 10.1016/j.ijbiomac.2020.05.083.

Zou, Y.F., Zhang, Y.Y., Zhu, Z.K., Fu, Y.P., Paulsen, B.S., Huang, C., et al. (2021). Characterization of inulin-type fructans from two species of Radix Codonopsis and their oxidative defense activation and prebiotic activities. *Journal of the Science of Food and Agriculture* 101(6)**,** 2491-2499. doi: 10.1002/jsfa.10875.
